# Ghardaqenoids A–F: Six New Diterpenoids from the South China Sea Soft Coral *Heteroxenia ghardaqensis* with Lipid-Lowering Activity via the Activation of the AMPK Signaling Pathway

**DOI:** 10.3390/md24010030

**Published:** 2026-01-08

**Authors:** Yue Zhang, Xin Han, Juan Wu, Shan Liu, Hongwei Zhang, Lili Zhao, Guoqiang Li

**Affiliations:** 1 Qingdao Medical College, Qingdao University, Qingdao 266071, China; zhangyueqy@163.com; 2 Marine Biomedical Research Institute of Qingdao, Qingdao 266003, China; 3 Key Laboratory of Marine Drugs, Chinese Ministry of Education, School of Medicine and Pharmacy, Ocean University of China, Qingdao 266003, China; 4 College of Basic Medicine, Jining Medical University, Jining 272067, China; 5 Marine Biomedical Research Institute of Qingdao, Ocean University of China, Qingdao 266003, China

**Keywords:** soft coral, *Heteroxenia ghardaqensis*, diterpenoids, absolute configurations, lipid-lowering activity

## Abstract

Six new diterpenoids, including two verticillane ghardaqenoids A–B (**1**–**2**) and four dolabellane ghardaqenoids C–F (**3**–**6**), were isolated from the soft coral *Heteroxenia ghardaqensis* collected in the South China Sea. The structures of ghardaqenoids A, D, and E (**1**, **4**, **5**) were determined by X-ray diffraction. Ghardaqenoids B, C, and F (**2**, **3**, **6**) were identified on the basis of NMR data, DP4+, and ECD spectral data. In particular, compound **6** exhibited strong in vitro lipid-lowering activity in free fatty acid (FFA)-induced HepG2 cells and liver organoids. Further mechanistic studies revealed that compound **6** regulated AMPK-related proteins and genes, thereby inhibiting the accumulation of triglycerides (TG) and total cholesterol (TC). These findings suggested that pharmacological AMPK activation serves as a promising role in lipid-lowering therapeutic strategies.

## 1. Introduction

Metabolic dysfunction-associated steatotic liver disease (MASLD), previously known as non-alcoholic fatty liver disease (NAFLD), has emerged as the most common chronic liver disease, with an overall prevalence of 38% and an increasing incidence each year [1,2,3]. MASLD can progress to severe hepatic conditions, including metabolic dysfunction-associated steatohepatitis (MASH), fibrosis, and hepatocellular carcinoma (HCC) [4]. Additionally, patients with MASLD have to face an elevated risk of developing type 2 diabetes, cardiovascular disease, chronic renal disease, and certain types of extrahepatic cancers [5,6]. The development and progression of MASLD are closely linked to hepatic lipid accumulation in hepatocytes and hepatosteatosis [7]. Hepatosteatosis occurs when there is an excess of lipids in hepatocytes due to factors such as enhanced de novo fatty acid production, increased lipid input from adipose tissue, and impaired lipid decomposition [8]. As a result, addressing the abnormalities of hepatic lipid metabolism may be an effective solution for the treatment of MASLD [9].

Recent studies suggest that adenosine monophosphate-activated protein kinase (AMPK) acts as a crucial metabolic regulator of glycolipid metabolism in hepatocytes [10,11,12,13]. Zhao et al. reported that liver-specific AMPK knockout exacerbated the pathological features in murine NASH models [14]. The activation of AMPK mitigates hepatic steatosis by suppressing fatty acid production [15] and promoting fatty acid oxidation [13]. Phosphorylated AMPK (p-AMPK) can inhibit the expression of key downstream genes involved in de novo fatty acid synthesis, including sterol regulatory element binding proteins (SREBPs), fatty acid synthase (FAS) and acetyl-CoA carboxylase(ACC) [16]. Additionally, low-dose sorafenib has recently been shown to effectively inhibit the progression of NASH in both mice and monkeys by the activation of AMPK [17]. Therefore, AMPK is crucial in lowering lipid accumulation and improving hepatic steatosis.

**Figure 1:**
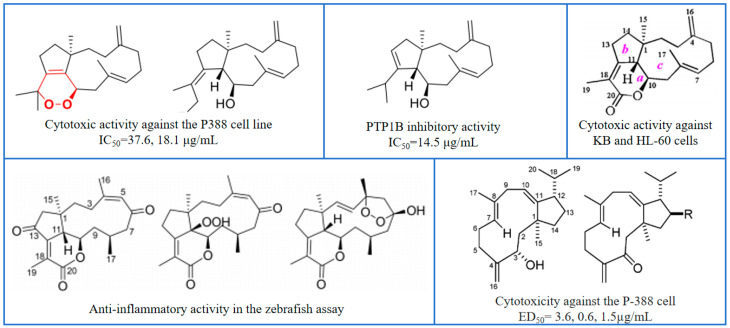
Some representative examples of active compounds.

The genus *Heteroxenia*, a type of marine soft coral, is taxonomically classified under the order Alcyonacea and the family Xeniidae. It is widely distributed along the Red Sea coast of Egypt, Mindoro Island in the Philippines, and other regions. In 1991, a research team led by D. Schlichter investigated the mutualistic symbiotic relationship between tropical soft corals and unicellular algae (genus *Symbiodinium*), which brought significant scientific attention to *Heteroxenia* species. Although research on this genus remains comparatively limited, several classes of secondary metabolites have been identified, including sterols [18], sesquiterpenes, and ceramides [19]. These compounds have been documented to exhibit a range of biological activities, such as cytotoxicity, anti-diabetic effects, antimicrobial properties, and anti-inflammatory activities [20] (Figure 1).

**Figure 2:**
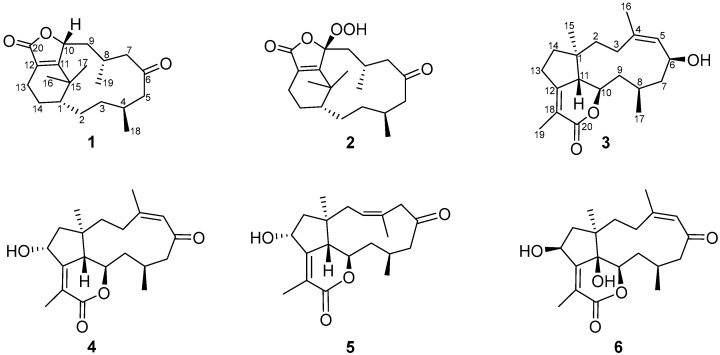
Proposed structures of ghardaqenoids A–F (**1**–**6**).

In the course of our continuous investigation into bioactive metabolites from South China Sea soft corals, two new verticillane diterpenoids, ghardaqenoids A–B (**1**–**2**), and four new dolabellane diterpenoids, ghardaqenoids C–F (**3**–**6**), were isolated from *Heteroxenia ghardaqensis*. The structures of all compounds (Figure 2) were elucidated by comprehensive spectroscopic analysis. Given the critical role of hepatic lipid dysregulation in metabolic dysfunction-associated fatty liver disease (MAFLD), and the established therapeutic potential of AMPK modulation in mitigating lipid accumulation and steatosis [21,22,23], the lipid-lowering activity of the isolated compounds was evaluated. Among them, compound **6** demonstrated a significant ability to reduce lipid deposition and ameliorate hepatic steatosis in both FFA-induced HepG2 cells and liver organoids. Mechanistic studies further revealed that this effect is primarily mediated through activation of the AMPK signaling pathway. These results identify compound **6** as a novel diterpenoid scaffold targeting AMPK, offering a promising lead structure for the development of new lipid-lowering therapeutics for MAFLD.

## 2. Results and Discussion

### 2.1. Structure Elucidation

**Figure 3:**
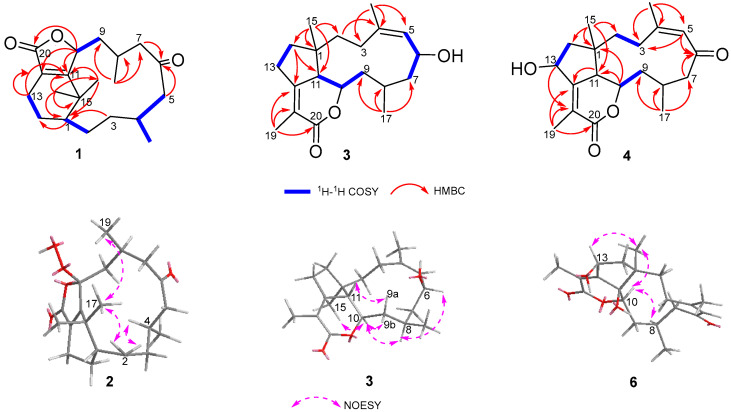
Key ^1^H-^1^H COSY, HMBC, and NOESY correlations of ghardaqenoids A–D and F (**1**–**4, 6**).

Ghardaqenoid A (**1**) was isolated as colorless crystals. Its molecular formula was established as C_20_H_30_O_3_ based on the (+)-HRESIMS ion observed at *m*/*z* 319.2268 [M + H]^+^ (calcd for C_20_H_31_O_3_, 319.2228), indicating six degrees of unsaturation. Interpretation of the ^1^H and ^13^C NMR data, assisted by the HSQC spectrum, disclosed 20 carbon resonances categorized as four methyls (*δ*_C_ 33.8, 25.1, 26.2, 22.3), seven methylenes (*δ*_C_ 53.4, 46.3, 37.4, 35.2, 33.2, 25.7, 16.6), four methines (*δ*_C_ 80.7, 43.3, 31.9, 24.6), and five quaternary carbons (including two carbonyls (*δ*_C_ 212.0, 173.3), two olefinic carbons (*δ*_C_ 169.1, 127.1), and one aliphatic quaternary carbon (*δ*_C_ 39.5)). These data, combined with the presence of two carbonyl groups and one double bond, suggested a tricyclic framework. COSY correlations delineated four spin systems: H-1/H-2, H_3_-18/H-4/H-5, H-9/H-10 and H-13/H-14 (Figure 3). These fragments were interconnected via HMBC correlations, enabling the assembly of the complete structure. Key HMBC interactions from the gem-methyl groups H_3_-17 (*δ*_H_ 1.21) and H_3_-16 (*δ*_H_ 1.14) to C-1, C-11, and C-15, along with correlations from H_2_-13 to C-11 and C-12, indicated a cyclohexene ring bearing a gem-dimethyl-substituted quaternary carbon adjacent to both a methine and an olefinic quaternary carbon (Table 1). Further HMBC correlations from H_3_-18 to C-3, C-4, and C-5; H_3_-19 to C-7, C-8, and C-9; H-10 to C-11; H_2_-7 to C-6; H_2_-5 to C-6; and H-3 to C-1, C-4 established a 12-membered lactone ring with methyl substituents at C-4 and C-8. The chemical shifts of C-20 (*δ*_C_ 173.3), C-12 (*δ*_C_ 127.1), C-11 (*δ*_C_ 169.1), and C-10 (*δ*_C_ 80.7), as well as HMBC correlations from H-10 to C-20 and C-12, were consistent with a γ-hydroxy-α,β-unsaturated five-membered lactone moiety. Thus, the gross structure of 1 was elucidated as depicted in Figure 2.

**Figure 4:**
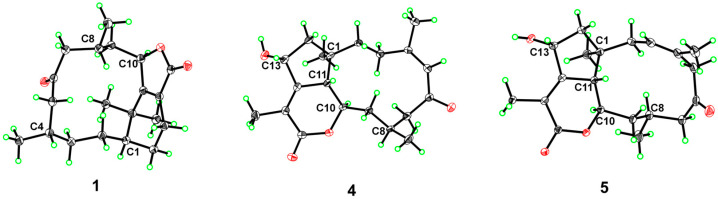
X-ray crystal structures of **1**, **4** and **5**.

Suitable single crystals of **1** for X-ray diffraction analysis were obtained by crystallization from methanol. The planar structure established above was unambiguously confirmed by X-ray crystallography using Cu Kα radiation (Figure 4). Furthermore, the experiment allowed the determination of the absolute configuration of **1**, which was assigned as 1*R*4*S*8*R*10*R* [CCDC deposition number: 2489353].

Ghardaqenoid B (**2**) was obtained as a colorless oil. Its molecular formula was established as C_20_H_30_O_5_ based on the (+)-HRESIMS peak observed at m/z 368.2431 [M + NH_4_]^+^ (calcd: 368.2431), indicating a mass increase of 32 units compared to **1** (**2** contains two additional oxygen atoms compared to **1**). Further structural analysis, supported by the characteristic chemical shift of C-10 (*δ*_C_ 114.9), indicates the presence of a peroxide bond attached to this carbon (Figure 2). In the NOESY spectrum, the correlations between Hₐ-2/H_3_-17, H_b_-2/H-4, and H_3_-17/H_3_-19 determine the relative configurations at positions C1, C4, and C8, which are consistent with the configurations observed in 1. The relative configuration at C-10 was unambiguously assigned using gauge-independent atomic orbital (GIAO) NMR chemical shift calculations at the PCM/B3LYP/6-311+G(d,p) level, followed by DP4+ probability analysis [14,15]. The absolute configuration of **2** was proposed to be 1*R*4*S*8*S*10*S* by comparing the experimental electronic circular dichroism (ECD) spectrum with data computed using time-dependent density functional theory (TDDFT) (Figure S2).

Ghardaqenoid C (**3**) was obtained as a colorless oil. The molecular formula C_20_H_30_O_3_ was verified by (+)-HRESIMS. The ^13^C NMR and HSQC spectra revealed 20 carbon signals, classified into four methyls, six methylenes, five methines, four quaternary carbons, and one ester carbonyl. The ^1^H NMR spectrum exhibited characteristic signals for an olefinic proton at *δ*_H_ 5.09 (1H, d, *J* = 7.4 Hz), an acetoxy-bearing methine at *δ*_H_ 4.32 (1H, m), two olefinic methyl groups at *δ*_H_ 1.83 (3H, s) and 1.72 (3H, s), and two additional methyl groups at *δ*_H_ 0.98 (3H, d, *J* = 5.8 Hz) and 0.85 (3H, s). Three of the six degrees of unsaturation were accounted for by two double bonds and one ester carbonyl, suggesting a tricyclic framework. COSY correlations defined three spin systems: H-5/H-6/H_2_-7, H_2_-9/H-10/H-11 and H_2_-13/H_2_-14. Key HMBC correlations from H_3_-17 to C-7, C-8, and C-9; H_3_-16 to C-3, C-4, and C-5; H_3_-15 to C-1, C-2, C-11, and C-14; H_2_-14 to C-11, C-12; H_2_-2 to C-4 and H-11 enabled the assembly of a bicyclic core structure. The chemical shift of C-6 (*δ*_C_ 67.5) is indicative of hydroxyl group substitution at this position. The presence of an α, β-unsaturated *δ*-lactone moiety was confirmed by HMBC correlations from H_3_-19 to C-12, C-18, and C-20, consistent with both the molecular formula and the deshielded resonance of H-10 (*δ*_H_ 4.32) (Figure 3). Based on NOESY correlations observed between H-10 and H-15; H_b_-9 and H-10; H_a_-9 and H-11; and H-8 with both H-10 and H-6, together with the large coupling constant between H-10 and H-11 (*J* = 9.1 Hz), the relative configuration of **3** was assigned as 1R*4*Z*6S*8R*10R*11S*. Furthermore, the absolute configuration was proposed to be 1*R*6*S*8*R*10*R*11*S* through TDDFT calculations of ECD performed at the RB3LYP/DGDZVP level (Figure S2).

Ghardaqenoid D (**4**) was obtained as colorless crystals, with HRESIMS analysis confirming its molecular formula as C_20_H_28_O_4_. Comprehensive NMR spectroscopic comparison revealed that **4** maintains substantial structural homology with **3** while exhibiting two distinct structural alterations: the significant downfield shift of C-6 from *δ*_C_ 67.5 to 206.7 indicates a carbonyl group at this position. Furthermore, the transformation of C-13 from a methylene to a methine carbon, accompanied by its characteristic chemical shift at *δ*_C_ 69.8, suggests hydroxyl group substitution at C-13. The configuration of 1*R*8*S*10*R*11*S*13*R* can be judged by X-ray diffraction (CCDC: 2478189).

Ghardaqenoid E (**5**) was obtained as colorless crystals and assigned the molecular formula C_20_H_28_O_4_ by HRESIMS, identical to that of **4**. The NMR spectra of **5** showed marked similarity to those of **4**, with the only significant difference being the positional shift of the double bond from Δ^4^ in 4 to Δ^3^ in **5**. Comparative structural analysis indicated that the primary distinction between these compounds lies in their chiral center configurations. The absolute configuration of **5** was unequivocally determined as 1*R*3*Z*8*S*10*R*11*S*13*R* by single-crystal X-ray diffraction analysis (CCDC: 2478190).

Ghardaqenoid F (**6**), C_20_H_28_O_5_, was established by HRESIMS and ^13^C NMR data (Table 2). Analysis of 1D and 2D NMR spectra revealed that **6** is structurally analogous to **4** but contains an additional oxygen atom. The chemical shift of C-11 (*δ*_C_ 76.7) indicated hydroxylation at this position, confirming the planar structure. NOESY correlations were used to determine the relative configuration: the cross-peak between H-5 and H-16 suggested a Z-configured double bond, while correlations of H-15 with H-10 and H-13, and of H-8 with H-10, indicated their co-facial orientation, supporting the assignment of 1R*8S*10R*13S*. The configuration at the quaternary center C-11 was designated as R* through DP4+ statistical analysis, resulting in the comprehensive relative configuration 1R*8S*10R*11R*13S*. The absolute configuration was proposed to be 1*R*8*S*10*R*11*R*13*S* through the comparison of experimental and TDDFT-calculated ECD spectra (Figure S2).

### 2.2. Biological Activity Assessment

#### 2.2.1. Effect of Compounds **1**–**6** on FFA-Induced Lipid Accumulation in HepG2 Cells

**Figure 5:**
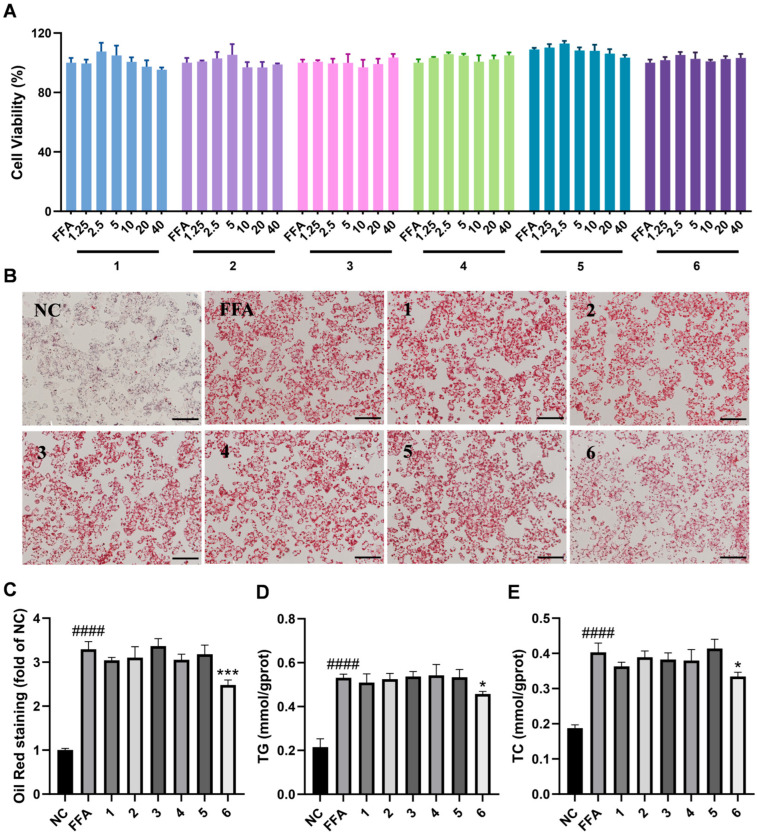
Screening lipid-lowering effects of compounds **1**–**6** in FFA-induced HepG2 cells. (**A**) Cytotoxic effects of compounds **1**–**6** on HepG2 cells were evaluated in the presence of FFAs. (**B**,**C**) Oil red O staining (scale bar = 100 μm) and semiquantitative analysis of the lipid content in HepG2 cells. (**D**) TG levels in HepG2 cells. (**E**) TC levels in HepG2 cells. Data are presented as the mean ± SD, *n* = 3. #### *p* <0.0001 vs. the normal control group; *** *p* < 0.001, * *p* < 0.05 vs. FFA-treated group.

HepG2 cell lines are extensively employed in drug screening and the investigation of the mechanisms behind MASLD owing to their stable phenotype and robust proliferative capacity [24,25]. In in vitro models, stimulation with FFAs can lead to the accumulation of total cholesterol (TC) and triglycerides (TG) in hepatocytes, subsequently inducing endoplasmic reticulum (ER) stress, inflammation, and cell death, which align closely with principal characteristics of MASLD [21,26]. Therefore, we performed the experiment using FFA-induced HepG2 cells to screen and evaluate the lipid-lowering efficacy of **6** new diterpenoids derived from soft coral. Following the establishment of the optimal concentration of FFAs (OA/PA  =  0.3 mM/0.15 mM as depicted in Figure S46), we evaluated the cytotoxicity of these compounds at concentrations ranging from 1.25 to 40 µM in both FFA-induced and normal HepG2 cells using the MTT test (Figure 5A and Figure S45). The results indicated that these compounds exhibited no significant cytotoxicity within this concentration range, suggesting that the dosages (0.45 mM of FFAs and 10 µM of compounds) used in subsequent studies were sufficiently biologically safe.

**Figure 6:**
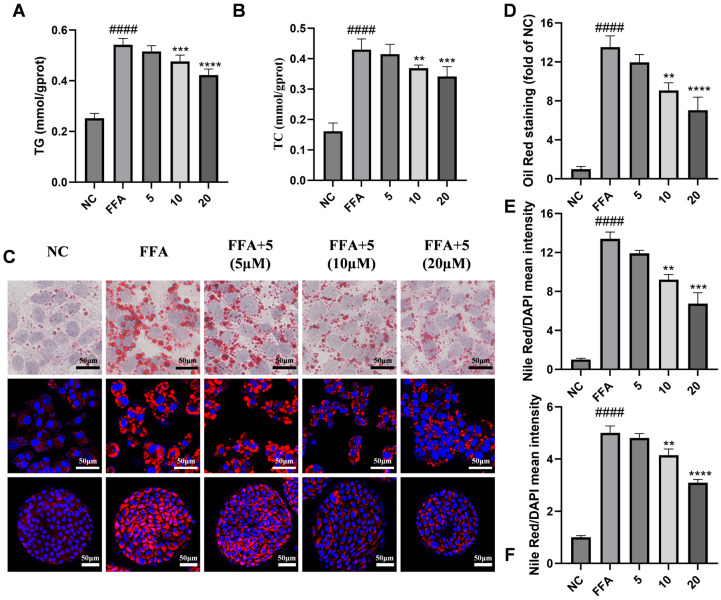
Compound **6** lowered lipid accumulation in FFA-induced HepG2 cells and FFA-induced steatosis organoids. (**A**,**B**) HepG2 cells were treated with 5–20 μM of compound **6** and 0.45 mM FFAs for 24 h. The intracellular TG and TC contents were measured using commercial kits. (**C**) Oil red O and Nile Red staining was performed on FFA-induced HepG2 cells and FFA-induced steatosis organoids. (**D**–**F**) Semiquantitative analysis of lipid content in FFA-induced HepG2 cells and steatosis organoids, respectively. Representative photographs of lipid droplets are presented (200× magnification). Semiquantitative analysis of the Oil red O stained area and Nile Red stained area was performed using ImageJ version 1.53k (NIH, Bethesda, MD, USA). Data are presented as the mean ± SD, *n* = 3. #### *p* < 0.0001 vs. the normal control group; **** *p* < 0.0001, *** *p* < 0.001, ** *p* < 0.01 vs. FFA-treated group.

Drug screening via Oil red O staining showed that compound **6** significantly decreased OA-induced lipid droplet accumulation in hepatocytes at a concentration of 10 μM (Figure 5B,C). Then, the inhibitory effect of compound **6** on lipid accumulation in HepG2 cells was further confirmed by measuring the contents of TG and TC (Figure 5D,E). After treatment with different concentrations of compound **6,** the levels of TG and TC in FFA-induced HepG2 cells were determined as shown in Figure 6A,B, suggesting that TG and TC contents were inhibited by compound **6** in a dose-dependent manner. Similarly, Oil red O and Nile red staining revealed a significant decrease in lipid accumulation in HepG2 cells treated with 5, 10, and 20 μM of compound **6** compared with the FFA-induced group (*p* < 0.001) in Figure 6C. The corresponding scanning images and the quantified fluorescence intensity data are presented in Figure 6D,E. To further evaluate the lipid-lowering effects in other in vitro models, we cultured human liver organoids and simulated lipid accumulation by FFAs for disease modeling of hepatic steatosis. Consistent with previous studies [27,28], Nile red staining data demonstrated that the quantity of lipid droplets was markedly greater in FFA-induced liver organoids than in those in the control group (Figure 6C,F), suggesting that FFAs could enhance the lipid accumulation of organoids. However, the lipid contents exhibited a significant decrease in spheroids treated with compound **6** in a dose-dependent manner. Taken together, these results suggest that compound **6** attenuates FFA-stimulated lipid accumulation in a safe concentration range in vitro.

#### 2.2.2. Effects of Compound **6** on the Expression of Lipid Metabolism-Related Proteins and Genes in FFA-Induced HepG2 Cells

**Figure 7:**
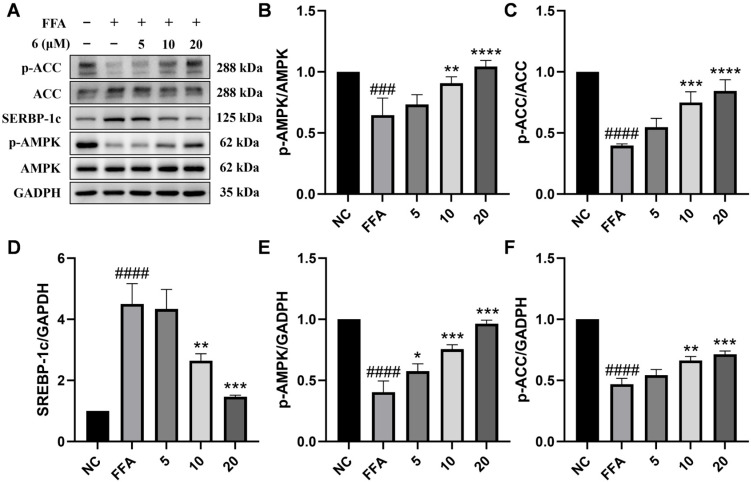
Effects of compound **6** on the expression of lipid metabolism-related proteins in FFA-induced HepG2 cells. (**A**–**F**) Representative Western blotting results and densitometric analysis of p-ACC/ACC, p-AMPK/AMPK, and SREBP-1c. Data are presented as the mean ± SD, *n* = 3. #### *p* < 0.0001, ### *p* < 0.001 vs. the normal control group; **** *p* < 0.0001, *** *p* < 0.001, ** *p* < 0.01, * *p* < 0.05 vs. FFA-treated group.

As a key cellular energy sensor, AMPK has been recognized as an important molecular target for the treatment of MASLD due to its function in integrating metabolic signals and controlling the balance between lipid synthesis and catabolism. Recent studies indicate that small-molecule drugs exhibit remarkable potential for activating the AMPK pathway and diminishing hepatic lipid deposition [20,28,29]. AMPK activation directly phosphorylates downstream significant effector molecules (ACC) to inhibit lipogenesis and promote fatty acid oxidation [29]. These findings provide a research guideline for investigating the regulation of metabolism mechanism of compound **6.** Therefore, this study used p-AMPK and p-ACC as the main detection indicators, based on a well-characterized molecular regulatory network to evaluate the metabolic regulatory mechanism of compound **6**. As illustrated in Figure 7, our results revealed that the activation of AMPK and phosphorylation of ACC were markedly suppressed in FFA-induced HepG2 cells, while the expression of SREBP-1c was elevated following FFA treatment, consistent with previous reports [30,31,32]. Compared to the FFA-treated group, the reduced levels of phosphorylated AMPK and ACC by FFAs were significantly reversed by compound **6**. Additionally, we further evaluated the mRNA level of SREBP-1c and observed a significant decrease after the treatment with compound **6** at 10 and 20 μM (Figure 7A–F).

**Figure 8:**
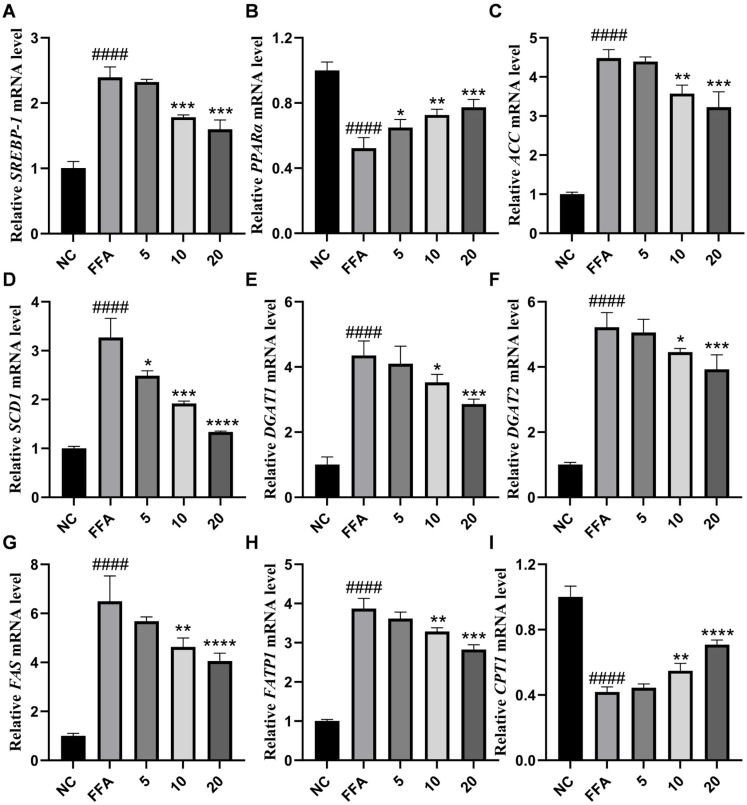
Effects of compound **6** on the mRNA expression of lipid metabolism-related genes in FFA-induced HepG2 cells. (**A**–**I**) The mRNA expression levels of *SREBP-1*, *PPARα*, *ACC*, *SCD1*, *DGAT1*, *DGAT2*, *FAS*, *FATP1*, and *CPT-1* from cell lysates were measured by RT-qPCR. All PCR reactions were normalized to *GAPDH* mRNA levels. Data are presented as the mean ± SD, *n* = 3. #### *p* < 0.0001 vs. the normal control group; **** *p* < 0.0001, *** *p* < 0.001, ** *p* < 0.01, * *p* < 0.05 vs. FFA-treated group.

Although the pivotal function of the AMPK pathway in lipid metabolism is well-established, substantial differences exist in the activation mechanisms of AMPK by distinct natural compounds and in the specificity of downstream regulation. To further explore lipid metabolism, the lipid metabolism-related genes in FFA-treated HepG2 cells were measured by RT-qPCR (Figure 8A–I). We found that compound **6** treatment reversed the down-regulation of the lipid oxidation genes *PPARα* and *CPT-1* in FFA-treated HepG2 cells, as well as the overexpression of *SREBP-1*, fat-related synthase genes (*ACC, SCD1, DGAT1, DGAT2, FAS*), and fatty acid transport proteins (*FATP1*). Taken together, these data strongly indicate that compound **6** inhibited lipid accumulation via activating the AMPK pathway in FFA-treated hepatocytes.

In the future, the scope of research requires further expansion. Multi-omics technologies, including transcriptomics and metabolomics, can help systematically assess the overall effects of compound **6** on the lipid metabolism profile and related signaling pathways in the liver. Additionally, it is essential to validate the regulatory expression of downstream target genes of *SREBP* (such as *FASN* and *SCD1*) by compound **6**, along with its impact on essential molecules involved in fatty acid oxidation (e.g., *CPT1* and *PPARα*), thereby establishing a more comprehensive molecular regulatory network. The development and therapeutic application of natural small molecule medications face numerous obstacles, such as low bioavailability, inadequate stability, and unclear target sites [32]. This work confirmed the activating effects of compound **6** on the AMPK pathway at the cellular level, thereby establishing a foundation for its subsequent in vivo activity assessment. Given the extensive expression of AMPK throughout many tissues and organs, it is imperative to assess the potential effects of compound **6** on systemic metabolism, including insulin sensitivity, adipose tissue distribution, liver steatosis, inflammatory infiltration, and fibrosis, thereby offering in vivo data to support its clinical safety. Furthermore, the structural modification of compound **6** will be carried out to deeply understand its bioavailability and targeting potential, as well as the pharmacokinetic characteristics, safety profile, and physicochemical properties. This could lead to the development of new candidate compounds for the formulation of safe and effective MASLD therapies.

## 3. Materials and Methods

### 3.1. General Experimental Procedures

Optical rotations were determined using a Jasco P-1020 polarimeter (Jasco, Tokyo, Japan). UV and circular dichroism spectra were obtained on a Jasco J-815 CD spectro-polarimeter (Jasco, Tokyo, Japan). NMR spectra were measured on JEOL JNMECZ 500 (^1^H, 500 MHz; ^13^C, 125 MHz) (JEOL, Tokyo, Japan) and 600 (^1^H, 600 MHz; ^13^C, 150 MHz) spectrometers (JEOL, Tokyo, Japan). *δ*_H_ 7.26 and *δ*_C_77.16 ppm were solvent signals of CDCl_3_. HRESIMS data were acquired on a Micromass Q-TOF Ultima GLOBAL GAA076LC (Waters, Shanghai, China). X-ray crystallographic data were obtained by a D8 Venture diffractometer (Bruker, Beijing, China) using Cu-Kα radiation. Semipreparative HPLC was performed with a Waters 1525 pump (Waters, Singapore), a YMC C18 or ODS column (10 × 250 mm, 5 μM), and a 2998 photodiode array detector. Chiral HPLC was performed using a chiral-phase column DAICEL IC-3. Silica gel (100–200 mesh, 300–400 mesh, and silica gel H, Qingdao, China) and Sephadex LH-20 (GE Healthcare Bio-Sciences AB, Danderyd, Sweden) were employed for column chromatography. For thin layer chromatography (TLC), silica gel plates (GF254, Qingdao, China) were utilized.

### 3.2. Soft Coral Material

Specimens of the soft coral *Heteroxenia ghardaqensis* were collected from the waters of the Xisha Islands in the South China Sea. The collection was conducted in strict compliance with the Specifications for Marine Surveys (GB/T 12763-2007) and relevant regulations on the protection of aquatic germplasm resources, using the manual diving collection method [33]. Healthy and intact coral colonies were selected for sampling. Immediately after collection, the specimens were processed on-site, quickly aliquoted into sterile cryovials, and cryopreserved in a portable −80 °C freezer. The morphological and molecular identification of this soft coral species was jointly completed by Dr. Leen van Ofwegen, an internationally renowned coral taxonomist from the Naturalis Biodiversity Center (Leiden, The Netherlands). Voucher specimens are currently deposited in the State Key Laboratory of Marine Drugs, Ocean University of China, P. R. China.

### 3.3. Extraction and Isolation

Frozen-preserved healthy soft coral samples (2 kg, wet weight) were selected and cut into uniform small pieces using sterile stainless steel scissors. The samples were extracted with methanol five times at room temperature (3 days per extraction). The solvent was removed under reduced pressure, and the residue was desalted with anhydrous methanol three times. The desalted residue (65 g) was subjected to silica gel vacuum column chromatography (CC) and eluted with a gradient of petroleum ether/acetone (20:1 to 1:1, *v*/*v*) followed by CH_2_Cl_2_/MeOH (10:1 to 0:1, *v*/*v*), yielding 6 fractions (Fr. 1–Fr. 6). Fractions 3–5 (150 mg) were purified by semi-preparative high-performance liquid chromatography (HPLC) using CH_3_OH/H_2_O (85:15, *v*/*v*) as the mobile phase at a flow rate of 2 mL·min^−1^, affording compound **3** (2.1 mg). Fractions 3–6 (180 mg) were purified by semi-preparative HPLC with MeCN/H_2_O (88:12, *v*/*v*) at 2 mL·min^−1^, obtaining compound **6** (2.2 mg). Fractions 4–8 (220 mg) were purified by semi-preparative HPLC using MeCN/H_2_O (83:17, *v*/*v*) at 2 mL·min^−1^, resulting in the isolation of compounds **1** (3.0 mg) and **2** (2.0 mg). Fractions 5–9 (340 mg) were purified by semi-preparative HPLC with CH_3_OH/H_2_O (80:20, *v*/*v*) at 2 mL·min^−1^, yielding compounds **4** (2.6 mg) and **5** (3.2 mg).

Ghardaqenoid A (**1**): colorless crystals; mp 152–154 °C; [*α*${]}_{D}^{25}$ −8.42 (c 0.5, MeOH); ^1^H NMR data is presented in Table 1; ^13^C NMR data is presented in Table 2; HRESIMS m/z 319.2268 [M + H] ^+^ (calcd. for C_20_H_31_O_3_, 319.2228).

Ghardaqenoid B (**2**): colorless oil; [*α*${]}_{D}^{25}$ −30.79 (c 0.5, MeOH); ^1^H NMR data is presented in Table 1; ^13^C NMR data is presented in Table 2; HRESIMS m/z 368.2431 [M + H]^+^ (calcd. for C_20_H_31_O_5_, 368.2431).

Ghardaqenoid C (**3**): colorless oil; [*α*${]}_{D}^{25}$ −61.40 (c 0.5, MeOH); ^1^H NMR data is presented in Table 1; ^13^C NMR data is presented in Table 2; HRESIMS m/z 323.2572 [M + H]^+^ (calcd. for C_20_H_35_O_3_, 323.2581).

Ghardaqenoid D (**4**): colorless crystals; mp 133.5–135.6 °C; [*α*${]}_{D}^{25}$ −31.2 (c 0.5, MeOH); ECD (1.0 mM, MeOH) λmax (Δε) 199 (4.27), 219 (−6.12), 238 (−1.07), 262 (−9.94); ^1^H NMR data is presented in Table 1; ^13^C NMR data is presented in Table 2; HRESIMS m/z 333.2060 [M + H]^+^ (calcd for C_20_H_29_O_4_, 333.2061).

Ghardaqenoid E (**5**): colorless crystals; mp 220.0–221.8 °C; [*α*${]}_{D}^{25}$ −244.4 (*c* 0.5, MeOH); ^1^H NMR data is presented in Table 1; ^13^C NMR data is presented in Table 2; HRESIMS m/z 333.2060 [M + H] ^+^ (calcd for C_20_H_29_O_4_, 333.2060).

Ghardaqenoid F (**6**): colorless oil; [*α*${]}_{D}^{25}$ −20.6 (c 0.10, MeOH); ECD (1.0 mM, MeOH) λmax (Δε) 199 (34.34), 226 (−66.67), 258 (−13.30); ^1^H NMR data is presented in Table 1; ^13^C NMR data is presented in Table 2; HRESIMS m/z 349.2010 [M + H]^+^ (calcd. for C_20_H_29_O_5_, 349.2013).

### 3.4. X-Ray Diffraction Data Analysis

Colorless single crystals (0.2 × 0.15 × 0.1 mm^3^) of compounds **1**, **4** and **5** were prepared via the vapor diffusion method from MeOH. X-ray single-crystal diffraction data were collected on a Bruker APEX-II CCD diffractometer using Cu Kα radiation as the X-ray source. Crystals were maintained at 150 K or 100 K with an Oxford Cryosystems low-temperature device. Crystal positioning and centering calibration were performed using a CCD camera, and data collection was carried out in the ω-scan mode. Raw diffraction data were processed with the Bruker APEX3 2022 software package to obtain independent diffraction reflections suitable for structural analysis. Crystal structure solution and refinement were accomplished using the Olex2 1.1 software suite: initial structure solution was performed with the SHELXT program (based on Intrinsic Phasing), while structural refinement was carried out using the SHELXL program (based on full-matrix least-squares minimization against F^2^). Structural validation was conducted via the PLATON program.

### 3.5. Quantum Chemical Calculations

Based on the initial structure of the compounds obtained from single-crystal X-ray diffraction, a full-geometry optimization without constraints was performed at the PCM/B3LYP/6-311+G (d, p) level of theory. The DP4+ probability analysis method was employed to evaluate the consistency between the calculated nuclear magnetic resonance (NMR) chemical shifts and the experimental data. Using the optimized stable configuration, electronic circular dichroism (ECD) spectrum calculations were conducted at the RB3LYP/DGDZVP level. The polarizable continuum model (PCM) was retained for simulating solvent effects with methanol as the solvent, ensuring consistency between the ECD calculation environment and the geometry optimization setup. All quantum mechanical calculations were implemented using the Gaussian 09 software package, while Spartan 14 software was utilized for initial configuration construction, visualization of calculation results, and molecular orbital analysis.

### 3.6. Cell Culture

HepG2 cells (ATCC, Manassas, VA, USA) were cultured in DMEM medium containing 10% Fetal bovine serum (ExCell Bio, Nanjing, China) and 1% Penicillin–Streptomycin (Solarbio Life Sciences, Beijing, China). Cells were maintained in a cell incubator with humidification in a stable environment, where the temperature was controlled at 37 °C and the CO_2_ concentration was maintained at 5%.

### 3.7. Cell Viability

Logarithmically growing HepG2 cells were inoculated into 96-well plates and cultured until they reached 80% confluence. To optimize the concentration and incubation time of free fatty acids (FFAs, oleate: palmitate at a 2:1 ratio), the plates were treated with 0–1.5 mM FFAs, or incubated for 0–48 h, respectively. After that, the cells were treated with compounds at concentrations ranging from 0 to 40 μM, along with 0.45 mM FFAs for 24 h. All experimental groups were tested with three independent replicates at each concentration. The viability of HepG2 cells was determined utilizing an MTT assay.

### 3.8. Cell FFA Treatment

HepG2 cells in the logarithmic growth phase were seeded onto glass coverslips in 24-well plates. After 12 h, when the cells reached 70–80% confluence, the medium was replaced with serum-free DMEM for a 12 h starvation. Subsequently, the cells were exposed to 0.45 mM FFAs in the presence or absence of compound **5** (5, 10, and 20 μM) for 24 h.

### 3.9. Cell Oil Red O Staining

Cells were fixed with 4% paraformaldehyde (PFA) fixative at room temperature, followed by aspiration of the fixative and three washes with PBS. Subsequently, the cells were rinsed with 60% isopropanol, and then stained with an Oil red O solution (Sigma-Aldrich, Burlington, MA, USA) by incubation at room temperature in the dark. After staining, the cells were washed with 60% isopropanol and gently rinsed with distilled water until the supernatant was colorless. The nuclei were counterstained with hematoxylin (Beijig Abgic Technology, Beijing, China). After mounting with glycerol gelatin, the stained cells were observed and imaged using an optical microscope. Semi-quantitative analysis of the stained images was conducted with ImageJ version 1.53k (NIH, Bethesda, MD, USA). All experiments were independently repeated in triplicate.

### 3.10. Cell Nile Red Staining

The cells were fixed with 4% PFA fixative for 20 min. Nile red (0.5 μg·mL^−1^, Thermo Fisher, Waltham, MA, USA) staining solution was added to ensure complete coverage of the cells, incubating for 30 min at room temperature. Subsequently, the sections were mounted with Antifade Mounting Medium with DAPI (Beyotime, Shanghai, Beijing) and further observed with an inverted fluorescence microscope and a laser-scanning confocal microscope (LSCM, Olympus, Tokyo, Japan). The experimental results were subjected to semiquantitative analysis via ImageJ version 1.53k (NIH, Bethesda, MD, USA).

### 3.11. Intracellular TG and TC Content Determination

Following induction with FFAs and treatment with compounds treatment, the primary culture medium was discarded, and the cells were subsequently washed three times with PBS. Then, the cells were digested with trypsin, centrifuged to discard the supernatant, and the cell pellet was retained. PBS was added to resuspend the cells, followed by ultrasonic disruption on ice to obtain a cell homogenate. The contents of intracellular triglycerides (TG) and total cholesterol (TC) were directly determined using a commercial kit (Jiancheng, Nanjing, China) following the manufacturer’s instructions.

### 3.12. Total RNA Isolation, Reverse Transcription (RT), and Quantitative Real-Time PCR (qPCR)

Total RNA was isolated from cells via the TRIzol lysis method, with its purity and concentration quantified using a Thermo Scientific NanoDrop One Spectrophotometer. Complementary DNA (cDNA) was synthesized through reverse transcription with Pre-mixed Solution for qPCR (Yeasen Biotechnology, Shanghai, China), wherein 1.0 μg of total RNA was reverse-transcribed into cDNA. The q-PCR reaction mixture (Yeasen Biotechnology, Shanghai, China) was prepared following the kit protocol, and the reaction plate was loaded onto a qPCR instrument. PCR amplification and subsequent analysis were performed using the ABI 7500 Real-Time PCR system (ABI, Indianapolis, IN, USA). The expression level of the target gene was normalized by *GAPDH*. All experiments were conducted in triplicate. The primer pairs utilized are provided in Table S4.

### 3.13. Western Blotting

The cells were lysed by RIPA lysis buffer (Beyotime, Shanghai, China) supplemented with Protease Phosphatase Inhibitor Mix (Solarbio Life Sciences, Beijing, China). After lysis on ice for 30 min, the cell lysate was centrifuged at 12,000× *g* for 10 min at 4 °C to obtain total cellular proteins. The concentration of total proteins was quantified using a BCA Protein Assay Kit, and the protein sample concentrations were adjusted to a consistent level based on the quantification results. Subsequently, 20 μg of protein per well was loaded onto a 10% sodium dodecyl sulfate–polyacrylamide gel electrophoresis (SDS-PAGE) gel for electrophoresis. The wet transfer method was used to transfer proteins from the gel to a polyvinylidene fluoride (PVDF) membrane (Sigma-Aldrich, Saint Louis, MO, USA). Then the PVDF membrane was blocked with 5% BSA at room temperature for 2 h, followed by three times washing with TBST. The membrane was then incubated overnight at 4 °C with diluted primary antibodies on a shaker, including phospho-AMPK (Cell Signaling Technology, Boston, MA, USA, 1:1000 dilution), AMPK (Starter, Hangzhou, China, 1:1000 dilution), phospho-ACC (Cell Signaling Technology, Boston, MA, USA, 1:1000 dilution), ACC (Cell Signaling Technology, Boston, MA, USA, 1:1000 dilution), SREBP-1 (Cell Signaling Technology, Boston, MA, USA, 1:1000 dilution), and GAPDH (Yeasen Biotechnology, Shanghai, China, 1:1000 dilution). On the following day, the primary antibody solution was discarded, and the membrane was washed three times with TBST. Specific proteins were detected by incubating the membrane with corresponding secondary antibodies on a shaker at room temperature for 2 h, including horseradish peroxidase (HRP)-conjugated goat anti-rabbit IgG (H+L) (ImmunoWay Biotechnology, TX, USA, 1:5000 dilution) or HRP-conjugated goat anti-mouse IgG (H+L) (ABclonal Technology, MA, USA, 1:5000 dilution). After secondary antibody incubation, the membrane was washed three times with TBST again. The developing solution was prepared by mixing equal volumes of Solution A and Solution B from the ECL Chemiluminescent Kit (Yeasen Biotechnology, Shanghai, China), which was then evenly added dropwise to the surface of the PVDF membrane. Protein bands were visualized and analyzed using a chemiluminescence imaging system (Tanon-5200, Qingdao, China). Densitometric analysis of the bands was performed using ImageJ version 1.53k (NIH, Bethesda, MD, USA). All experiments were independently repeated three times.

### 3.14. Organoid Culture and Steatosis Induction

Human hepatocyte organoids were established and induced to steatosis according to a previous study. Briefly, liver tissues were first minced with a scalpel, then digested with a pre-prepared digestive solution (Sigma-Aldrich). The isolated hepato-cytes were collected by low-speed centrifugation, and the cell pellet was resuspended in AdvDMEM+++ and seeded in 25 μL per droplet of BME (Cultrex) suspension (2:1 BME: AdvDMEM+++) per well of a 48-well plate. AdDMEM/F-12 medium contained 1× GlutaMAX, 10 mM HEPES, and 100 U mL^−1^ penicillin/streptomycin solution (all provided by Gibco, Grand Island, NY, USA). Organoids were maintained in HEP medium. The culture medium was supplemented with 15% RSPO1-conditioned medium (MCE), 1×N2 Supplement, 1× B-27 Supplement Minus Vitamin A (Gibco), 10 mM nicotinamide (Sigma-Aldrich), 1.25 mM N-acetyl-l-cysteine (Sigma-Aldrich), 50 ng·mL^−1^ hEGF, 0.1 μg·mL^−1^ Wnt3A, 50 ng·mL^−1^ hFGF10, 25 ng·ml^−1^ hHGF, (all provided by MCE, Milan, Italy), 10 nM gastrin (Tocris, Bristol, UK), 5 μM A 83-01 (MCE), 5 μM Forskolin, 10 μM Y-27632 (MCE) and 100 μg·mL^−1^ primocin (InvivoGen, San Diego, CA, USA). The culture medium was changed every 3–4 days. Organoids became visible 2–14 days after seeding and were monitored daily using a bright-field microscope to identify growing organoids. Organoids were digested and dissociated every 7–10 days based on their density and size. The general splitting ratio was 1:4 to 1:8, depending on the growth rate of the donor organoid line. The steatotic organoids were induced by exogenous FFAs (0.5 mM) for 3 days. To evaluate the lowering activity, the steatotic organoids were treated with compound **5** at different concentrations for 7 days and analyzed by evaluating the lipid droplet fluorescence of all organoids.

### 3.15. Statistical Analysis

Experimental data are presented as the mean ± standard deviation (SD). Statistical analyses were performed utilizing GraphPad Prism 10.0. Differences among groups were evaluated by one-way analysis of variance (ANOVA). A *p*-value of less than 0.05 was considered statistically significant, while a *p*-value of less than 0.01 was regarded as highly statistically significant.

## 4. Conclusions

In conclusion, six previously undescribed diterpenoids, designated ghardaqenoids A–F (**1**–**6**), were isolated from the methanol extract of 2 kg (wet weight) of frozen soft coral *Heteroxenia ghardaqensis*, yielding a total mass of approximately 15.1 mg (~0.00075% *w*/*w* relative to fresh material) through sequential desalting, silica gel vacuum chromatography, and semi-preparative HPLC. Among them, compounds **1** and **2** possess a verticillane skeleton, while **3**–**6** belong to the dolabellane class, with their absolute configurations established by single-crystal X-ray diffraction, TDDFT, and ECD calculations. Cytotoxicity screening revealed that these compounds were non-cytotoxic, and notably, preliminary mechanistic evaluation identified compound **6** as a modulator of hepatic lipid metabolism through AMPK-ACC pathway activation, positioning it as a promising lead candidate for the intervention of metabolic dysfunction-associated steatotic liver disease (MASLD). Future work should prioritize in-depth mechanistic studies, in vivo validation, and optimization of drug-like properties to advance this scaffold toward novel clinical therapies.

## Figures and Tables

**Table 1:** ^1^H NMR spectroscopic data for compounds **1**–**6** in 500 MHz in CDCl_3_.

No.	1	2	3	4	5	6
	*δ*_H_ (*J* in Hz)	*δ*_H_ (*J* in Hz)	*δ*_H_ (*J* in Hz)	*δ*_H_ (*J* in Hz)	*δ*_H_ (*J* in Hz)	*δ*_H_ (*J* in Hz)
1	1.46, m	1.42, br				
2	0.96, m; 1.18, d (5.5)	0.84, m; 1.08, m	2.07, m; 1.30, m	1.80, m; 1.54, m	2.64, m; 2.16, m	2.02, m; 1.62, m
3	1.48, m; 1.65, m	1.50, m; 1.62, m	2.27, m; 1.83, m	2.26, m	5.28, d (15.6)	2.33, m; 2.02, m
4	1.73, m	1.77, m				
5	2.09, dd (4.5, 17.0); 2.66, dd (4.5, 17.0)	2.11, dd (4.5, 17.0); 2.65, dd (4.5, 17.0)	5.09, d (7.4)	5.95, s	3.87, d (18.6); 2.46, d (18.6)	5.98, m
6			4.59, m			
7	2.20, d (8.0); 2.30, br, d (3.5)	2.27, m; 2.34, m	1.45, m	1.80, m; 1.54, m	2.62, m; 2.11, m	2.47, m; 2.23, m
8	1.61, m	1.64, m	1.49, m	2.28, m	2.47, m	2.13, m
9	1.97, m; 2.14, br	1.93, m; 2.39, dd (8.0, 15.0)	2.21, m; 1.78, m	1.92, m; 1.64, m	1.75, m; 1.56, m	1.82, m; 1.58, m
10	5.18, s		4.32, m	4.43, m	4.37, m	4.37, d (8.4)
11			2.70, d (9.1)	2.35, m	2.57, m	2.47, m; 2.23, m
13	2.28, m	2.31, m	2.47, m; 2.38, m	4.80, d (8.0)	4.80, d (10.8)	4.73, m
14	1.59, m; 2.14, m	1.58, m; 2.16, m	1.83, m; 1.45, m	1.94, m; 1.66, m	2.28, m; 1.65, m	2.00, m
15			0.85, s	1.08, s	1.20, s	0.80, s
16	1.14, s	1.25, s	1.72, s	1.83, d (1.3)	1.90, s	1.80, s
17	1.21, s	1.29, s	0.98, d (5.8)	1.18, d (7.2)	1.09, d (9.6)	1.19, d (6.8)
18	1.09, d (7.2)	1.09, d (7.0)				
19	1.15, d (7.0)	1.20, d (6.5)	1.83, s	2.03, m	2.03, m	1.94, s

**Table 2:** ^13^C NMR spectroscopic data for compounds **1**–**6** in 125 MHz in CDCl_3_.

No.	1	2	3	4	5	6
	*δ*_C_, Type	*δ*_C_, Type	*δ*_C_, Type	*δ*_C_, Type	*δ*_C_, Type	*δ*_C_, Type
1	43.3, CH	44.4, CH	45.9, C	44.6, C	45.1, C	46.3, C
2	33.2, CH_2_	33.4, CH_2_	39.6, CH_2_	37.9, CH_2_	37.6, CH_2_	27.7, CH_2_
3	35.2, CH_2_	35.8, CH_2_	26.3, CH_2_	28.1, CH_2_	122.2, CH	28.2, CH_2_
4	31.9, CH	31.9, CH	138.8, C	148.1, C	132.1, C	147.4, C
5	46.3, CH_2_	46.8, CH_2_	129.0, CH	130.0, CH	42.9, CH_2_	131.2, CH
6	212.0, C	211.9, C	67.5, CH	206.7, C	207.6, C	208.6, C
7	53.4, CH_2_	53.4, CH_2_	45.9, CH_2_	49.0, CH_2_	55.9, CH_2_	49.0, CH_2_
8	24.6, CH	27.0, CH	24.1, CH	30.9, CH	25.8, CH	34.7, CH
9	37.4, CH_2_	38.4, CH_2_	46.2, CH_2_	41.0, CH_2_	43.3, CH_2_	35.4, CH_2_
10	80.7, CH	114.9, C	77.8, CH	80.0, CH	78.3, CH	83.8, CH
11	169.1, C	164.6, C	51.7, CH	53.2, CH	51.0, CH	76.7, C
12	127.1, C	131.3, C	160.1, C	158.6, C	160.4, C	158.1, C
13	16.6, CH_2_	16.7, CH_2_	26.9, CH_2_	69.8, CH	69.8, CH	68.9, CH
14	25.7, CH_2_	25.3, CH_2_	36.9, CH_2_	50.1, CH_2_	48.8, CH_2_	49.6, CH_2_
15	39.5, C	39.7, C	23.8, CH_3_	21.1, CH_3_	25.4, CH_3_	21.4, CH_3_
16	33.8, CH_3_	37.9, CH_3_	23.1, CH_3_	23.5, CH_3_	24.8, CH_3_	22.3, CH_3_
17	25.1, CH_3_	24.0, CH_3_	21.8, CH_3_	18.1, CH_3_	20.3, CH_3_	23.2, CH_3_
18	22.3, CH_3_	22.4, CH_3_	119.8, C	124.1, C	125.0, C	126.0, C
19	26.2, CH_3_	25.6, CH_3_	12.5, CH_3_	13.0, CH_3_	13.0, CH_3_	12.2, CH_3_
20	173.3, C	170.4, C	165.8, C	165.6, C	166.4, C	166.2, C

## Data Availability

Data are contained within the article or Supplementary Materials; further inquiries can be directed to the corresponding authors.

## Supplementary Materials

The following supporting information can be downloaded at: https://www.mdpi.com/article/10.3390/md24010030/s1. Tables S1–S3: X-ray crystallographic analyses of **1**, **4** and **5**; Table S4: Sequence of primers for qRT-PCR; Figure S1: Detailed DP4+ probability (calculated at PCM/b3lyp/6-311+G(d,p) level) for compound **2**; Figure S2: Experimental and calculated ECD spectra of **2**, **3** and **6**; Figures S3–S44: HRESIMS, 1D and 2D NMR spectra of all new compounds **1**–**6**; Figure S45: Cytotoxic effects of compounds on HepG2 cells; Figure S46: Effects of (Left) FFAs concentration (48 h), (Right) FFAs stimulation duration.
