# Supplementary material for: Ghardaqenoids A–F: Six New Diterpenoids from the South China Sea Soft Coral Heteroxenia ghardaqensis with Lipid-Lowering Activity via the Activation of the AMPK Signaling Pathway
Source: Mar Drugs. 2026 Jan 8;24(1):30. doi: 10.3390/md24010030 (PMC12843345; doi:10.3390/md24010030)
Supplement: Supplementary file 1 [file marinedrugs-24-00030-s001.zip › Supporting Information.pdf]

Supporting Information

Ghardaqenoids A–F, Six New Diterpenoids from the South China Sea soft coral *Heteroxenia ghardaqensis* with Lipid-lowering Activity *via* the activation of AMPK signaling pathway

Yue Zhang [a,b,†], Xin Han [c,d,†], Juan Wu [b], Shan Liu [b], Hongwei Zhang [b,e], Lili Zhao [b,e,*], Guoqiang Li [c *]

[a] Qingdao Medical College, Qingdao University, Qingdao 266071, People's Republic of China

[b] Marine Biomedical Research Institute of Qingdao, Qingdao 266003, People's Republic of China

[c] Key Laboratory of Marine Drugs, Chinese Ministry of Education, School of Medicine and Pharmacy, Ocean University of China, Qingdao 266003, People's Republic of China

[d] College of Basic Medicine, Jining Medical University, Jining 272067, People's Republic of China

[e] Marine Biomedical Research Institute of Qingdao, Ocean University of China, Qingdao 266003, People's Republic of China

[†] Yue Zhang and Xin Han contributed equally to this work

Corresponding Authors

*E-mail: zhaolili@ouc.edu.cn

liguoqiang@ouc.edu.cn

List of Supplementary Information

Table S1. X-ray diffraction analysis of compound **1**. 4
Table S2. X-ray diffraction analysis of compound **4**. 5
Table S3. X-ray diffraction analysis of compound **5**. 6
Table S4. Sequence of primers for qRT-PCR. 7
Figure S1. Detailed DP4+ probability (calculated at PCM/b3lyp/6-311+G(d,p) level) for compound **2**. 8
Figure S2. Experimental and calculated ECD spectra of **2, 3** and **6**. 9
Figure S3. HRESIMS data of ghardaqenoid A (**1**). 10
Figure S4. ^{1}H NMR spectrum of ghardaqenoid A (**1**) in $CDCl_3$ (500 MHz). 10
Figure S5. ^{13}C NMR spectrum of ghardaqenoid A (**1**) in $CDCl_3$ (125 MHz). 11
Figure S6. ^{1}H-^{1}H COSY spectrum of ghardaqenoid A (**1**) in $CDCl_3$. 12
Figure S7. HSQC spectrum of ghardaqenoid A (**1**) in $CDCl_3$. 12
Figure S8. HMBC spectrum of ghardaqenoid A (**1**) in $CDCl_3$. 13
Figure S9. NOESY spectrum of ghardaqenoid A (**1**) in $CDCl_3$. 13
Figure S10. HRESIMS data of ghardaqenoid B (**2**). 14
Figure S11. ^{1}H NMR spectrum of ghardaqenoid B (**2**) in $CDCl_3$ (500 MHz). 15
Figure S12. ^{13}C NMR spectrum of ghardaqenoid B (**2**) in $CDCl_3$ (125 MHz). 15
Figure S13. ^{1}H-^{1}H COSY spectrum of ghardaqenoid B (**2**) in $CDCl_3$. 16
Figure S14. HSQC spectrum of ghardaqenoid B (**2**) in $CDCl_3$. 16
Figure S15. HMBC spectrum of ghardaqenoid B (**2**) in $CDCl_3$. 17
Figure S16. NOESY spectrum of ghardaqenoid B (**2**) in $CDCl_3$. 17
Figure S17. HRESIMS data of ghardaqenoid C (**3**). 18
Figure S18. ^{1}H NMR spectrum of ghardaqenoid C (**3**) in $CDCl_3$ (500 MHz). 19
Figure S19. ^{13}C NMR spectrum of ghardaqenoid C (**3**) in $CDCl_3$ (125 MHz). 19
Figure S20. ^{1}H-^{1}H COSY spectrum of ghardaqenoid C (**3**) in $CDCl_3$. 20
Figure S21. HSQC spectrum of ghardaqenoid C (**3**) in $CDCl_3$. 20
Figure S22. HMBC spectrum of ghardaqenoid C (**3**) in $CDCl_3$. 21
Figure S23. NOESY spectrum of ghardaqenoid C (**3**) in $CDCl_3$. 21
Figure S24. HRESIMS data of ghardaqenoid D (**4**). 22
Figure S25. ^{1}H NMR spectrum of ghardaqenoid D (**4**) in $CDCl_3$ (500 MHz). 23
Figure S26. ^{13}C NMR spectrum of ghardaqenoid D (**4**) in $CDCl_3$ (125 MHz). 23
Figure S27. ^{1}H-^{1}H COSY spectrum of ghardaqenoid D (**4**) in $CDCl_3$. 24
Figure S28. HSQC spectrum of ghardaqenoid D (**4**) in $CDCl_3$. 24
Figure S29. HMBC spectrum of ghardaqenoid D (**4**) in $CDCl_3$. 25
Figure S30. NOESY spectrum of ghardaqenoid D (**4**) in $CDCl_3$. 25
Figure S31. HRESIMS data of ghardaqenoid E (**5**). 26
Figure S32. ^{1}H NMR spectrum of ghardaqenoid E (**5**) in $CDCl_3$ (500 MHz). 27
Figure S33. ^{13}C NMR spectrum of ghardaqenoid E (**5**) in $CDCl_3$ (125 MHz). 27
Figure S34. ^{1}H-^{1}H COSY spectrum of ghardaqenoid E (**5**) in $CDCl_3$. 28
Figure S35. HSQC spectrum of ghardaqenoid E (**5**) in $CDCl_3$. 28
Figure S36. HMBC spectrum of ghardaqenoid E (**5**) in $CDCl_3$. 29
Figure S37. NOESY spectrum of ghardaqenoid E (**5**) in $CDCl_3$. 29
Figure S38. HRESIMS data of ghardaqenoid F (**6**). 30
Figure S39. ^{1}H NMR spectrum of ghardaqenoid F (**6**) in $CDCl_3$ (500 MHz). 31

Figure S40. ^{13}C NMR spectrum of ghardaqenoid F (**6**) in $CDCl_3$ (125 MHz). 31
Figure S41. ^1H-^1H COSY spectrum of ghardaqenoid F (**6**) in $CDCl_3$. 32
Figure S42. HSQC spectrum of ghardaqenoid F (**6**) in $CDCl_3$. 32
Figure S43. HMBC spectrum of ghardaqenoid F (**6**) in $CDCl_3$. 33
Figure S44. NOESY spectrum of ghardaqenoid F (**6**) in $CDCl_3$. 33
Figure S45. Cytotoxic effects of compounds on HepG2 cells. 34
Figure S46. Effects of (Left) FFAs concentration (48 h), (Right) FFAs stimulation duration (0.45 mM FFAs; 300μM oleic acid + 150 μM palmitic acid) 34

Table S1. X-ray diffraction analysis of compound **1**.

Identification code	cu_0913_6_0m
Empirical formula	$C_{20}H_{30}O_3$
Formula weight	318.44
Temperature/K	150.00
Crystal system	monoclinic
Space group	$P2_1$
a/Å	9.6985(3)
b/Å	8.4181(3)
c/Å	10.8402(3)
α/°	90
β/°	97.0690(10)
γ/°	90
Volume/Å^3	878.30(5)
Z	2
ρ_{calc}g/cm^3	1.204
μ/mm^{-1}	0.622
F(000)	348.0
Crystal size/mm^3	0.2 × 0.15 × 0.1
Radiation	CuKα (λ = 1.54178)
2Θ range for data collection/°	8.218 to 144.236
Index ranges	$-11 \leq h \leq 10$, $-10 \leq k \leq 10$, $-13 \leq l \leq 10$
Reflections collected	5224
Independent reflections	3132 [R_{int} = 0.0221, R_{sigma} = 0.0317]
Data/restraints/parameters	3132/1/212
Goodness-of-fit on F^2	1.094
Final R indexes [I>=2σ (I)]	R_1 = 0.0610, wR_2 = 0.1406
Final R indexes [all data]	R_1 = 0.0613, wR_2 = 0.1410
Largest diff. peak/hole / e Å^{-3}	0.35/-0.22
Flack parameter	0.13(7)

Table S2. X-ray diffraction analysis of compound **4**.

Identification code	Ck5321
Empirical formula	$C_{20}H_{28}O_4$
Formula weight	332.42
Temperature/K	170(2)
Crystal system	monoclinic
Space group	$P2_1$
a/Å	9.1852(4)
b/Å	8.7620(3)
c/Å	11.2872(4)
α/°	90
β/°	103.276(2)
γ/°	90
Volume/$Å^3$	884.13(6)
Z	2
$\rho_{calc}g/cm^3$	1.249
μ/mm^{-1}	0.686
F(000)	360.0
Crystal size/mm^3	0.09 × 0.07 × 0.06
Radiation	CuKα (λ = 1.54178)
2Θ range for data collection/°	9.894 to 149.484
Index ranges	$-11 \leqslant h \leqslant 11, -10 \leqslant k \leqslant 10, -14 \leqslant l \leqslant 13$
Reflections collected	21080
Independent reflections	3587 [R_{int} = 0.0524, R_{sigma} = 0.0345]
Data/restraints/parameters	3587/1/222
Goodness-of-fit on F^2	1.067
Final R indexes [I>=2σ (I)]	R_1 = 0.0596, wR_2 = 0.1651
Final R indexes [all data]	R_1 = 0.0620, wR_2 = 0.1670
Largest diff. peak/hole / e $Å^{-3}$	0.46/-0.30
Flack parameter	0.08(9)

Table S3. X-ray diffraction analysis of compound **5**.

Identification code	Ck6342
Empirical formula	$C_{20}H_{30}O_5$
Formula weight	350.44
Temperature/K	170(2)
Crystal system	monoclinic
Space group	$P2_1$
a/Å	9.1035(4)
b/Å	6.3047(3)
c/Å	17.2126(7)
α/°	90
β/°	105.247(2)
γ/°	90
Volume/Å^3	953.14(7)
Z	2
ρ_{calc}g/cm^3	1.221
μ/mm^{-1}	0.700
F(000)	380.0
Crystal size/mm^3	0.16 × 0.02 × 0.02
Radiation	CuKα (λ = 1.54178)
2Θ range for data collection/°	5.322 to 149.962
Index ranges	-11 ≤ h ≤ 11, -7 ≤ k ≤ 7, -21 ≤ l ≤ 21
Reflections collected	18218
Independent reflections	3839 [R_{int} = 0.0435, R_{sigma} = 0.0315]
Data/restraints/parameters	3839/1/234
Goodness-of-fit on F^2	1.038
Final R indexes [I>=2σ (I)]	R_1 = 0.0426, wR_2 = 0.1161
Final R indexes [all data]	R_1 = 0.0449, wR_2 = 0.1176
Largest diff. peak/hole / e Å^{-3}	0.22/-0.17
Flack parameter	0.01(9)

Table S4. Sequence of primers for qRT-PCR.

Species	Name	Primer	Sequence (5′–3′)
Homo	*ACC*	FP	GAACCATCTCCCTTGGCCC
		RP	CAAGGCCAAGCCATCCTGTA
Homo	*SCD1*	FP	CTTGCGATATGCTGTGGTGC
		RP	AAGTTGATGTGCCAGCGGTA
Homo	*FAS*	FP	GGACCCTCCTACCTCTGGTT
		RP	ACCTGGAGGACAGGGCTTAT
Homo	*DGAT1*	FP	CCCCAACAAGGACGGAGAC
		RP	AACCGGGCATTGCTCAAGAT
Homo	*DGAT2*	FP	TCTGGGAGATGGGGAGTGG
		RP	CACCAGCTGGATGGGAAAGT
Homo	*CPT-1*	FP	GCAGCGTTCTTTGTGACGTT
		RP	AGGAGTGTTCAGCGTTGAGG
Homo	*GAPDH*	FP	CTTTGTCAAGCTCATTTCCTGG
		RP	TCTTCCTCTTGTGCTCTTGC
Homo	*FATP1-F*	FP	ACGCGATATACCAGGAGCTG
		RP	ACGCGATATACCAGGAGCTG
Homo	*SREBP-1*	FP	ACTTCTGGAGGCATCGCAAGCA
		RP	AGGTTCCAGAGGAGGCTACAAG
Homo	*PPARα*	FP	TTCGCAATCCATCGGCGAG
		RP	CCACAGGATAAGTCACCGAGG

Functional		Solvent?		Basis Set		Type of Data	
B3LYP		PCM		6-31+G(d,p)		Unscaled Shifts	
		DP4+	100.00%	0.00%	-	-	-
Nuclei	sp2?	xperimenta	Isomer 1	Isomer 2	Isomer 3	Isomer 4	Isomer 5
C	x	131.25	141.141456	140.59213			
C		16.68	21.7127359	23.2934424			
C		25.29	31.1709783	26.2378287			
C		44.41	50.3446978	46.1041326			
C		39.69	48.1542176	45.0650185			
C	x	164.64	179.661817	186.610053			
C		114.85	122.636677	121.798783			
C	x	170.08	178.108777	178.484738			
C		38.43	39.7746622	37.9607378			
C		26.95	34.7314687	33.2191694			
C		53.19	56.540539	51.6157285			
C	x	211.88	226.978692	224.152698			
C		46.77	48.5405036	57.4277287			
C		31.91	38.4829588	35.8220691			
C		24.04	36.6448081	22.4419947			
C		33.41	37.4208128	32.0331989			
C		35.78	37.9206805	41.2229047			
C		32.92	25.8658961	25.6127553			
C		25.64	26.8760781	24.3420379			
C		22.39	23.4295406	28.246298			
H		2.31	2.30552152	2.42264503			
H		2.31	2.31975607	2.40177409			
H		1.58	1.58770656	1.64050205			
H		2.16	2.30328049	2.02568205			
H		1.42	1.42292712	0.7598926			
H		2.26	2.41318816	2.96906198			

H		2.26	2.41318816	2.96906198
H		1.94	2.01984567	2.21789888
H		1.64	1.48555328	2.31740923
H		2.34	1.88259683	2.28034769
H		2.27	2.41032054	2.28339316
H		2.65	2.97793113	2.04497411
H		2.11	2.1956651	2.53217827
H		1.77	1.58798229	2.37489274
H		1.25	1.72164463	1.48417656
H		1.25	1.19523664	0.94276656
H		1.25	1.05710438	1.24543283
H		1.08	1.163467	1.16676669
H		0.84	0.97360757	1.6461968
H		1.62	1.37986109	1.49705899
H		1.5	2.02117558	1.27007127
H		1.29	1.16235573	0.83299958
H		1.29	1.57152405	0.85694198
H		SSs	1.15725925	1.30864297
H		1.2	1.56262467	0.92384475
H		1.2	0.87789272	1.02879947
H		1.2	1.25095799	1.33916784
H		1.09	0.98125748	0.97178976
H		1.09	1.72310002	0.9758515
H		1.09	0.9027927	1.04528433

Functional	Solvent?		Basis Set		Type of Data	
B3LYP	PCM		6-31+G(d,p)		Unscaled Shifts	
	Isomer 1	Isomer 2	Isomer 3	Isomer 4	Isomer 5	Isomer 6
sDP4+ (H data)	100.00%	0.00%	-	-	-	-
sDP4+ (C data)	100.00%	0.00%	-	-	-	-
sDP4+ (all data)	100.00%	0.00%	-	-	-	-
uDP4+ (H data)	100.00%	0.00%	-	-	-	-
uDP4+ (C data)	99.98%	0.02%	-	-	-	-
uDP4+ (all data)	100.00%	0.00%	-	-	-	-
DP4+ (H data)	100.00%	0.00%	-	-	-	-
DP4+ (C data)	100.00%	0.00%	-	-	-	-
DP4+ (all data)	100.00%	0.00%	-	-	-	-

Figure S1. Detailed DP4+ probability (calculated at PCM/b3lyp/6-311+G(d,p) level) for compound **2**.

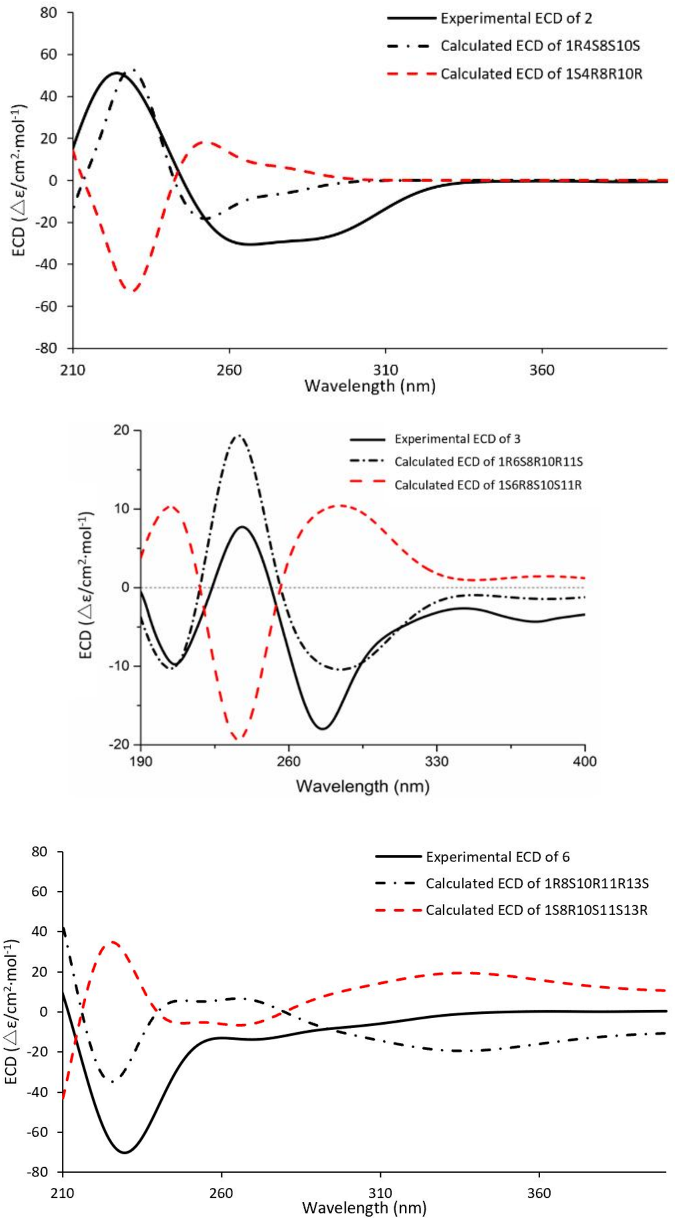

Figure S2. Experimental and calculated ECD spectra of **2, 3** and **6**.

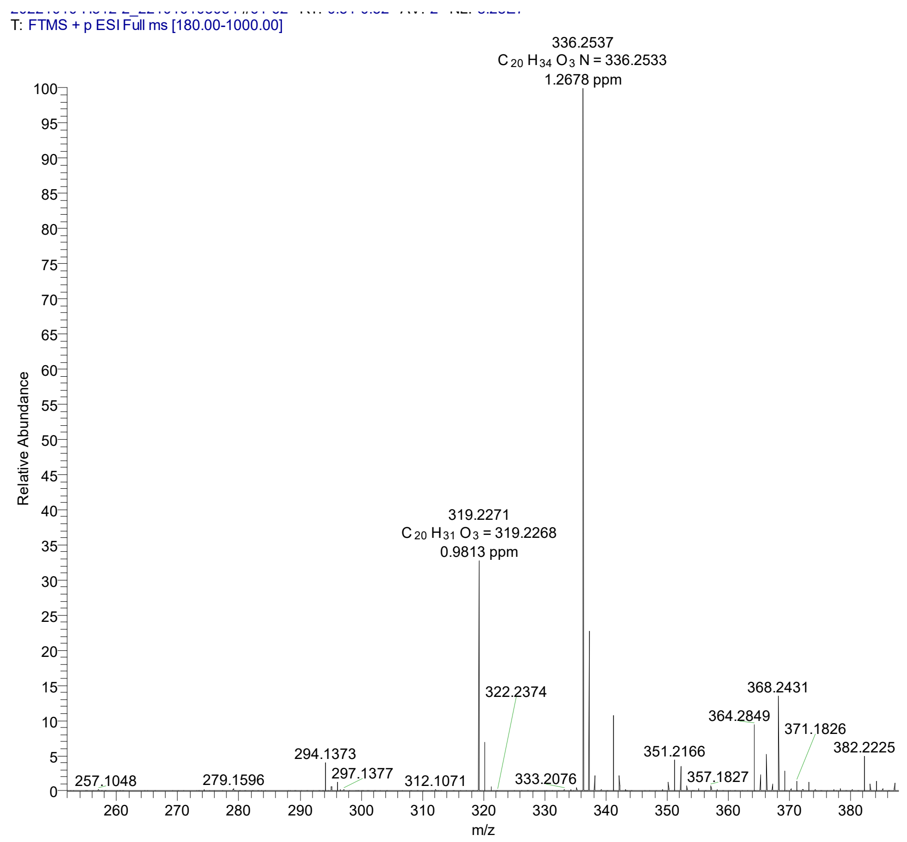

Figure S3. HRESIMS data of ghardaqenoid A (**1**).

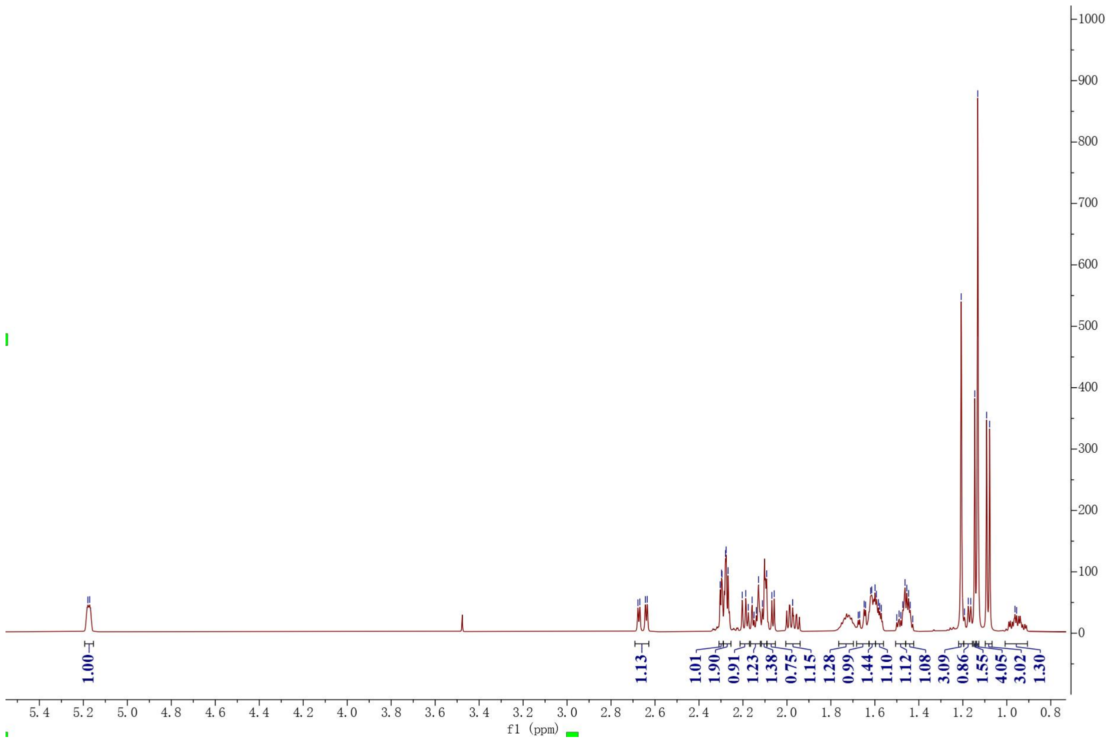

Figure S4. ^{1}H NMR spectrum of ghardaqenoid A (**1**) in $CDCl_3$ (500 MHz).

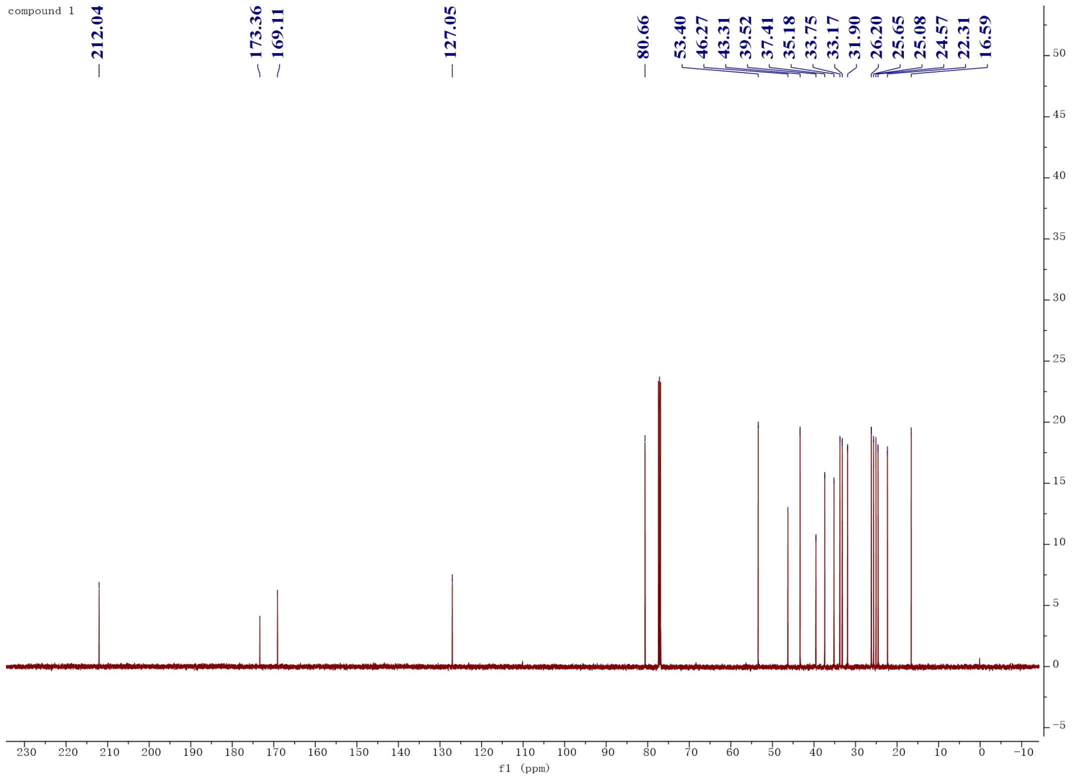

Figure S5. ^{13}C NMR spectrum of ghardaqenoid A (**1**) in $CDCl_3$ (125 MHz).

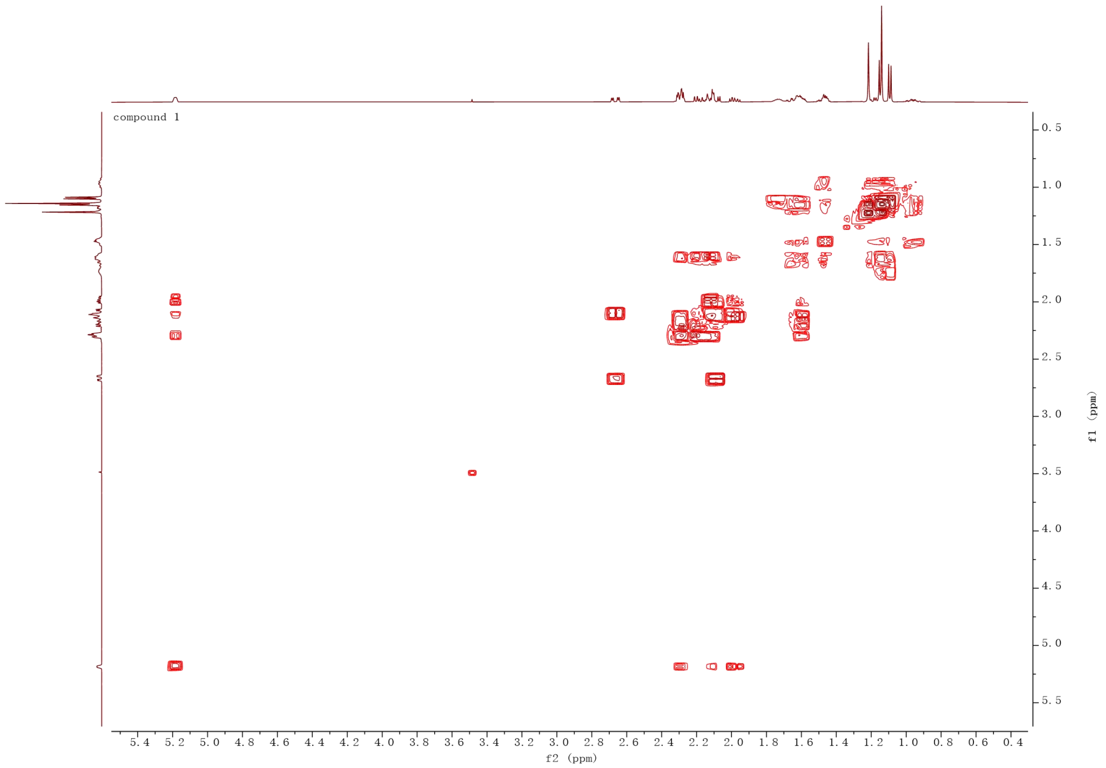

Figure S6.^{1}H-^{1}H COSY spectrum of ghardaqenoid A (**1**) in $CDCl_3$.

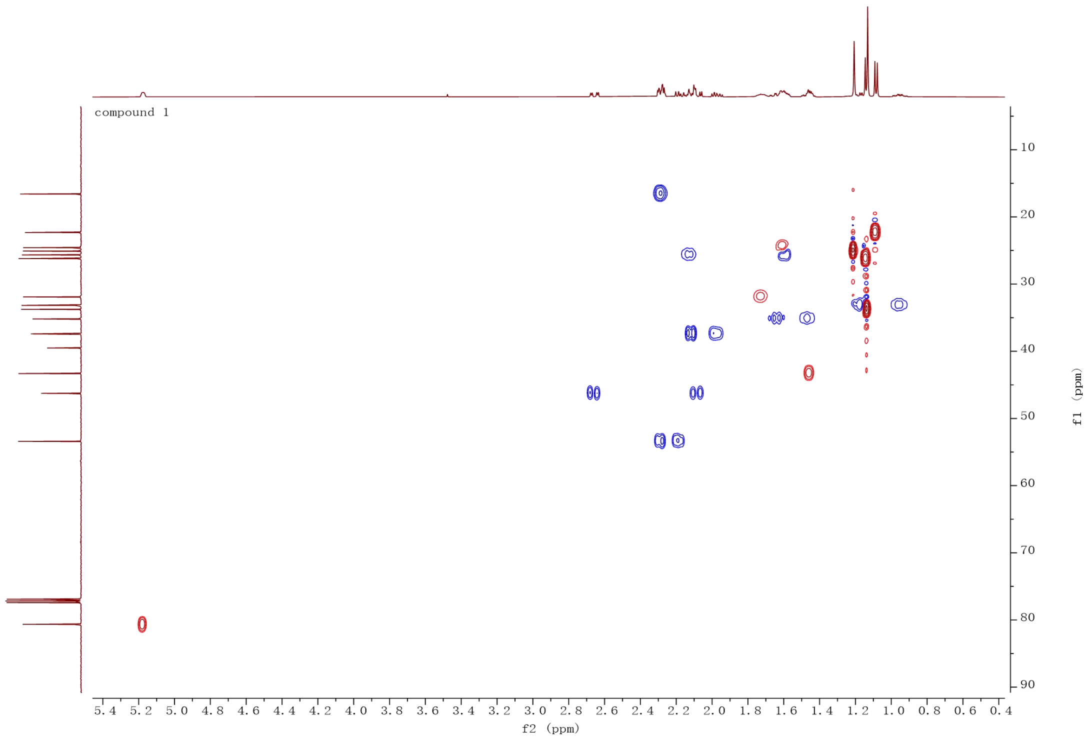

Figure S7. HSQC spectrum of ghardaqenoid A (**1**) in $CDCl_3$.

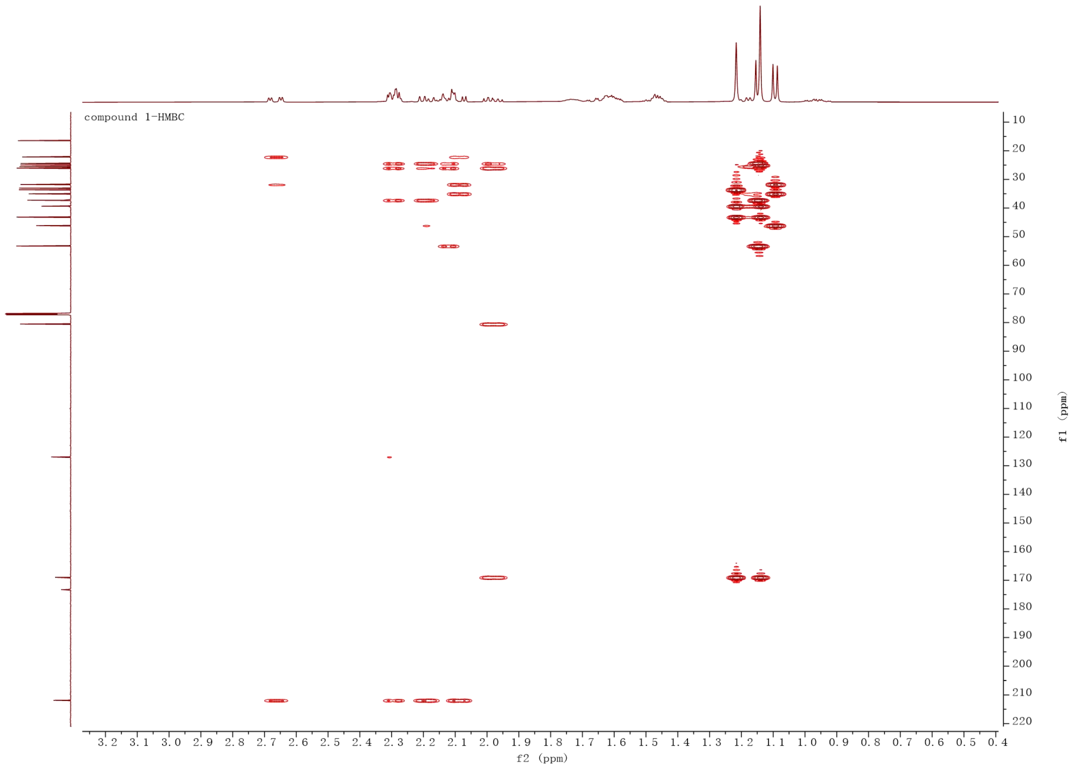

Figure S8. HMBC spectrum of ghardaqenoid A (**1**) in $CDCl_3$.

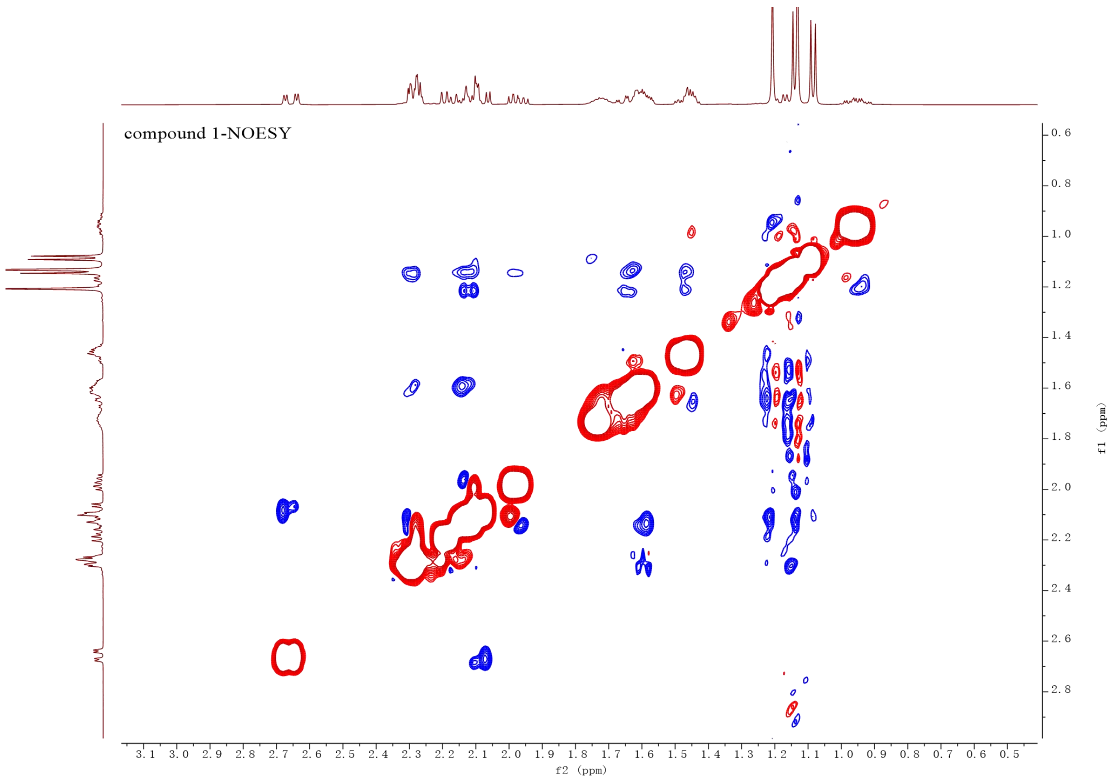

Figure S9. NOESY spectrum of ghardaqenoid A (**1**) in $CDCl_3$.

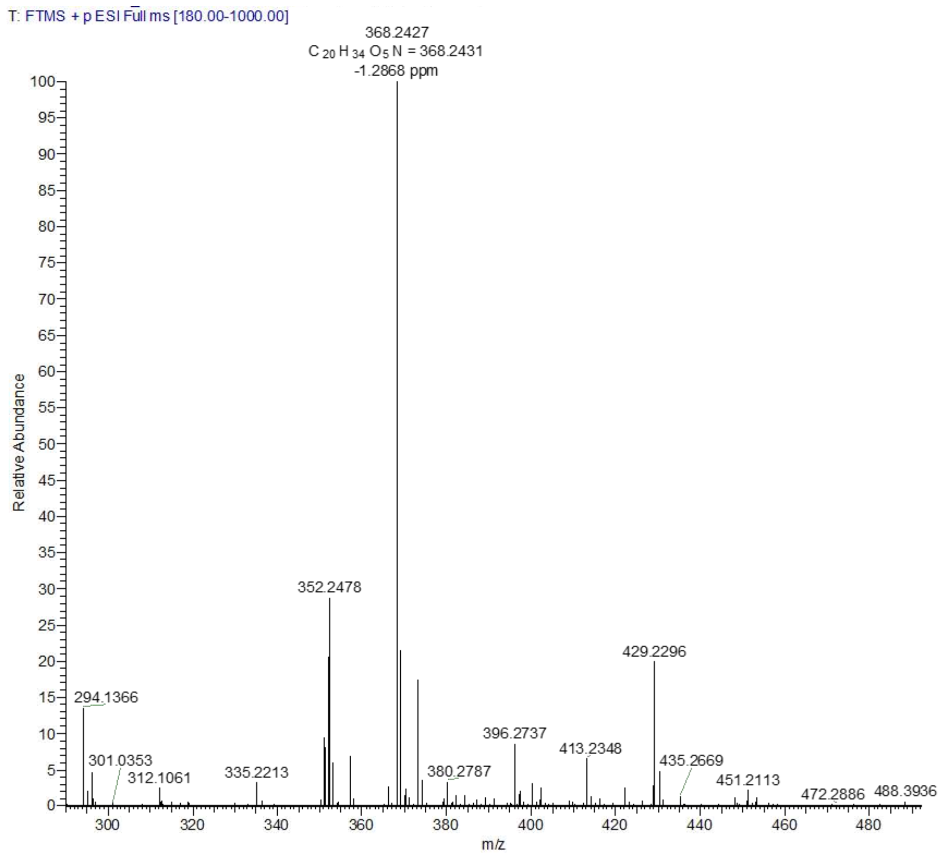

Figure S10. HRESIMS data of ghardaqenoid B (**2**).

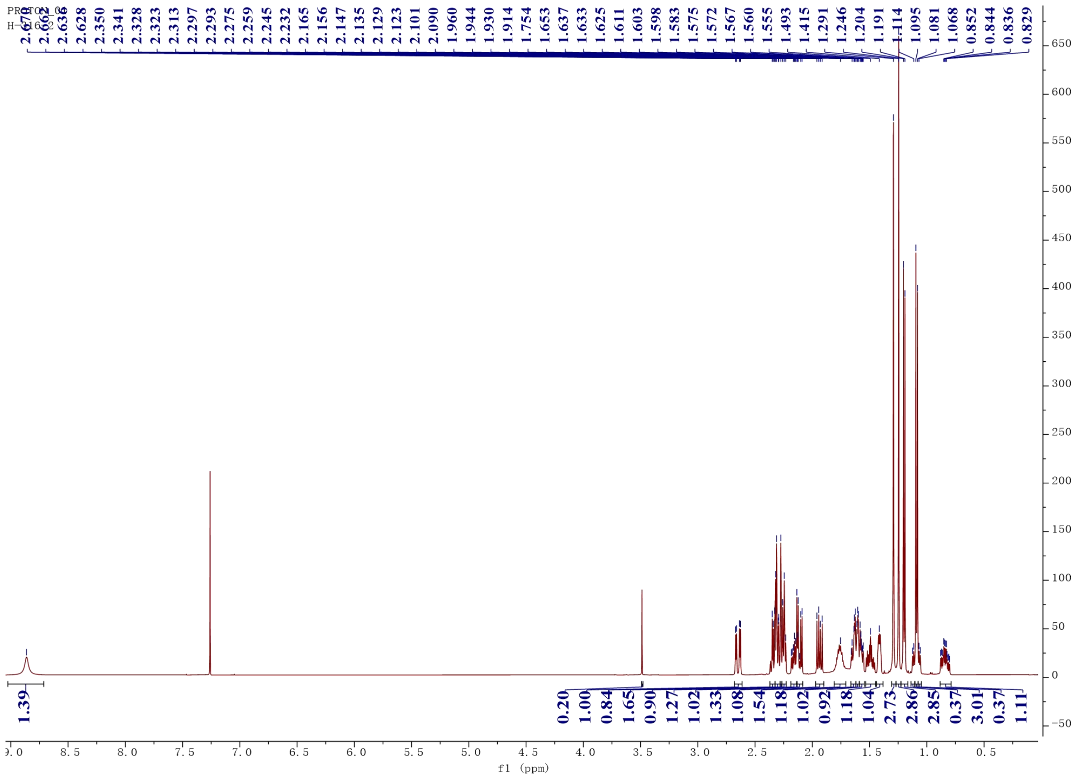

Figure S11. ^{1}H NMR spectrum of ghardaqenoid B (**2**) in $CDCl_3$ (500 MHz).

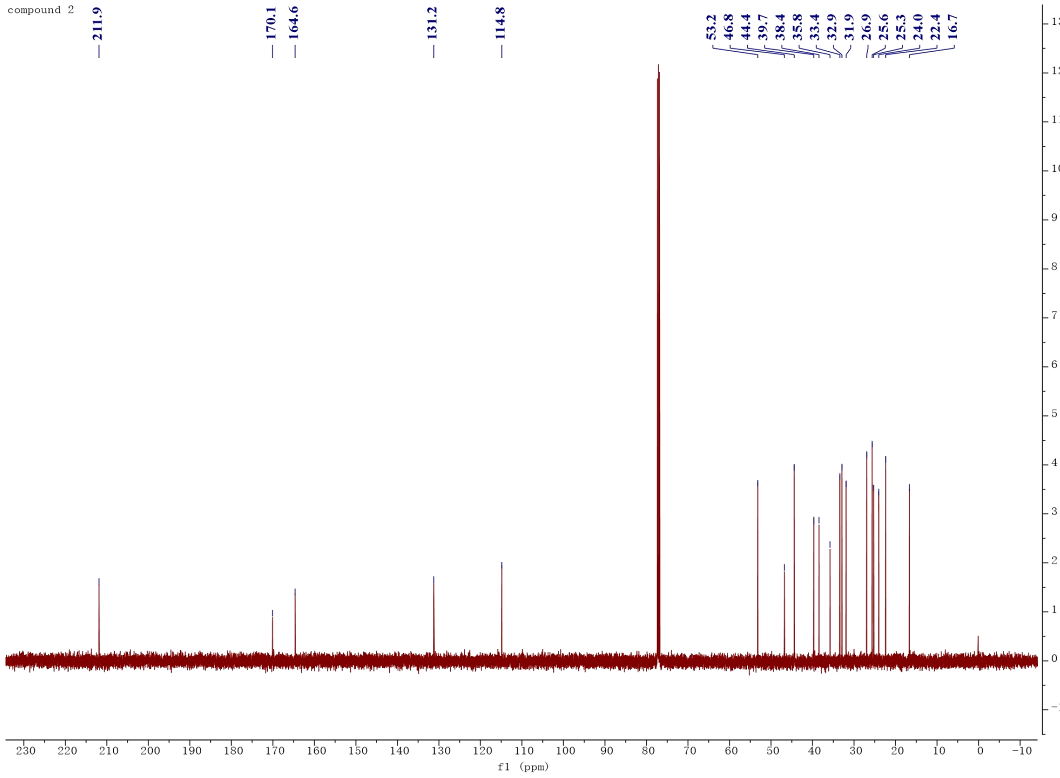

Figure S12. ^{13}C NMR spectrum of ghardaqenoid B (**2**) in $CDCl_3$ (125 MHz).

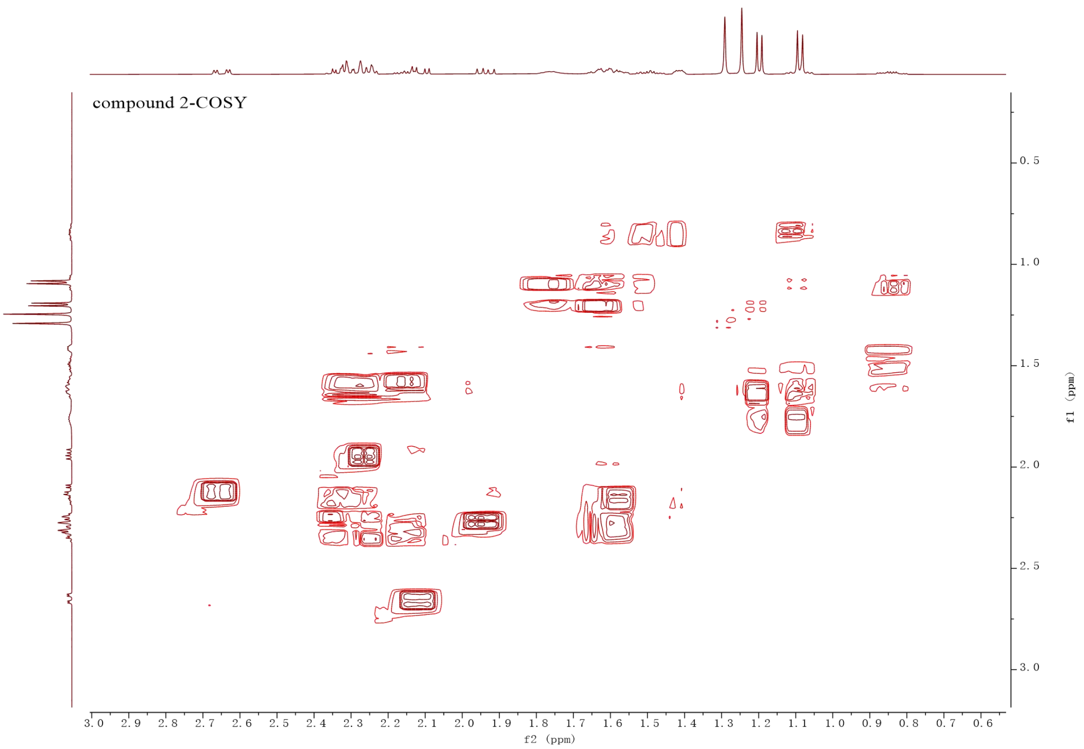

Figure S13.^{1}H-^{1}H COSY spectrum of ghardaqenoid B (**2**) in $CDCl_3$.

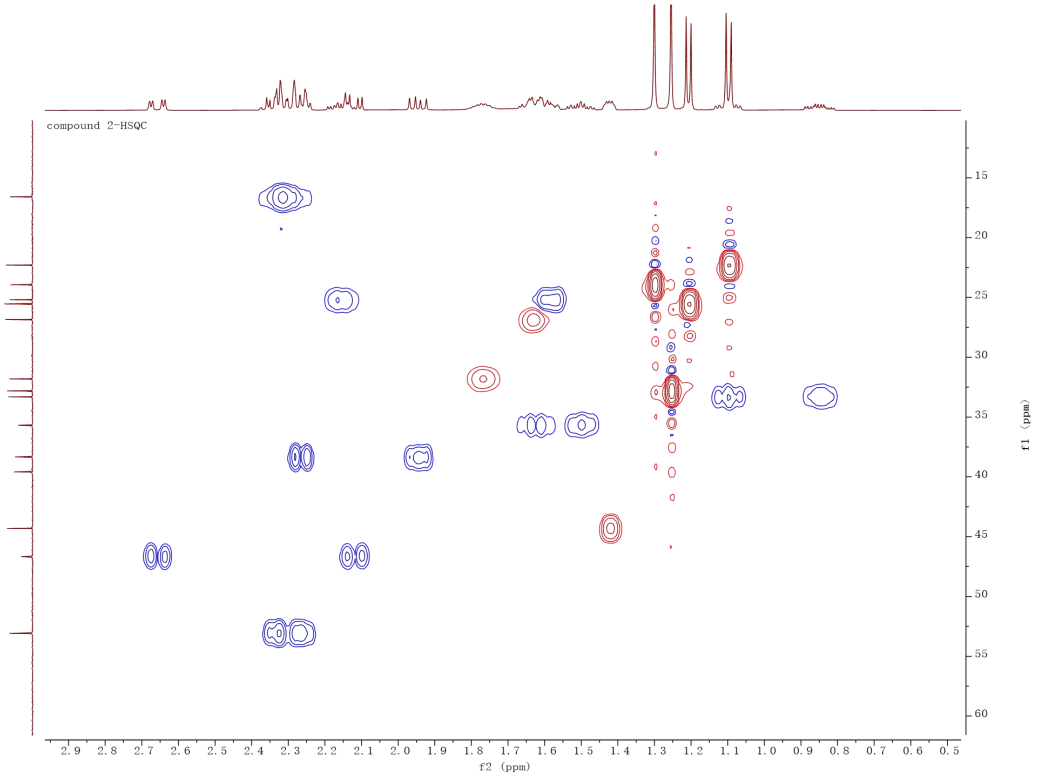

Figure S14.HSQC spectrum of ghardaqenoid B (**2**) in $CDCl_3$.

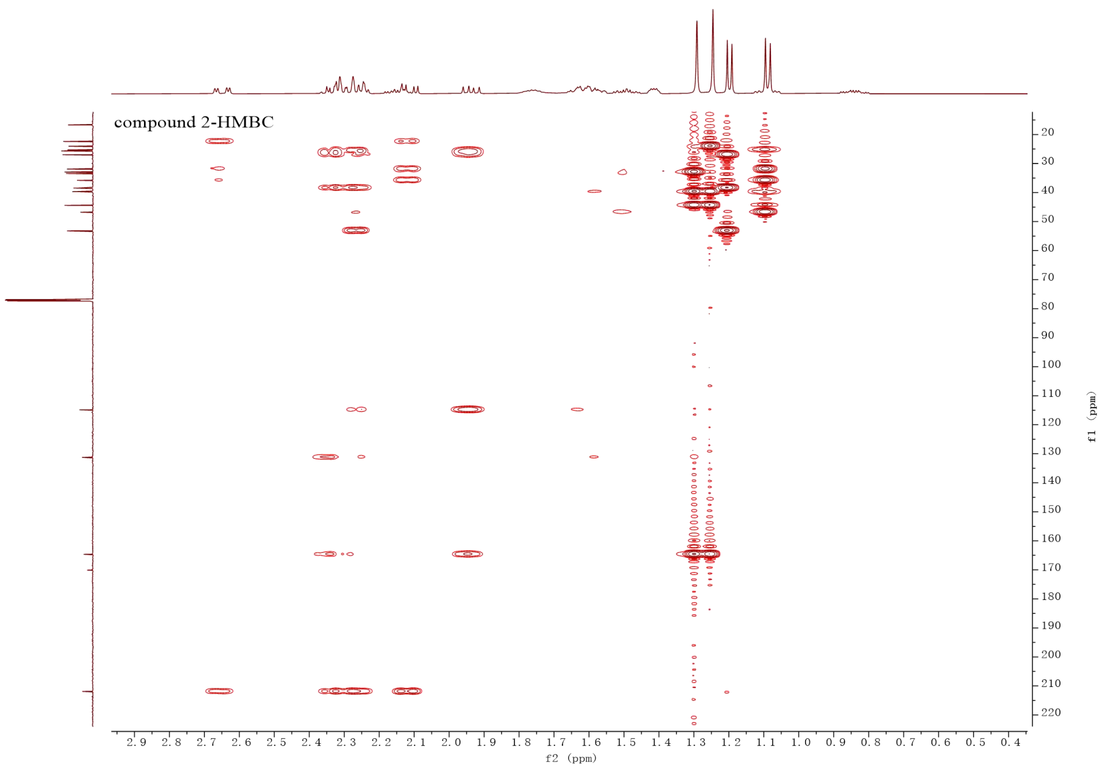

Figure S15. HMBC spectrum of ghardaqenoid B (**2**) in $CDCl_3$.

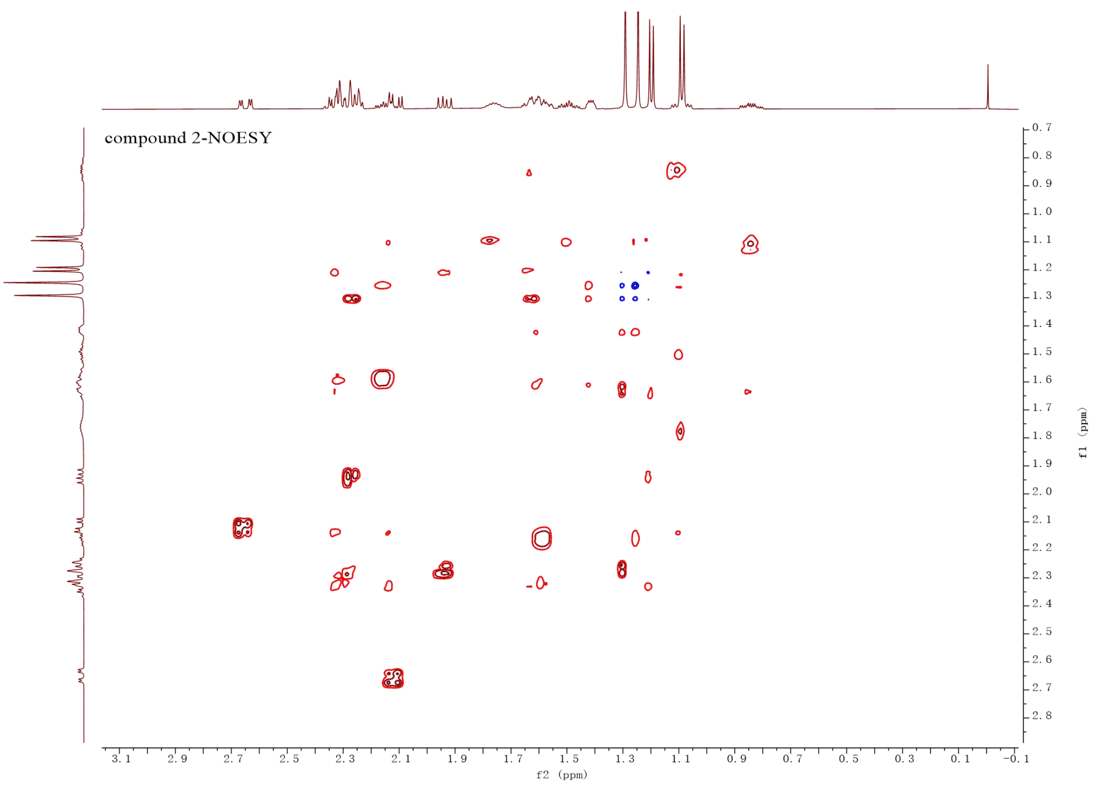

Figure S16. NOESY spectrum of ghardaqenoid B (**2**) in $CDCl_3$.

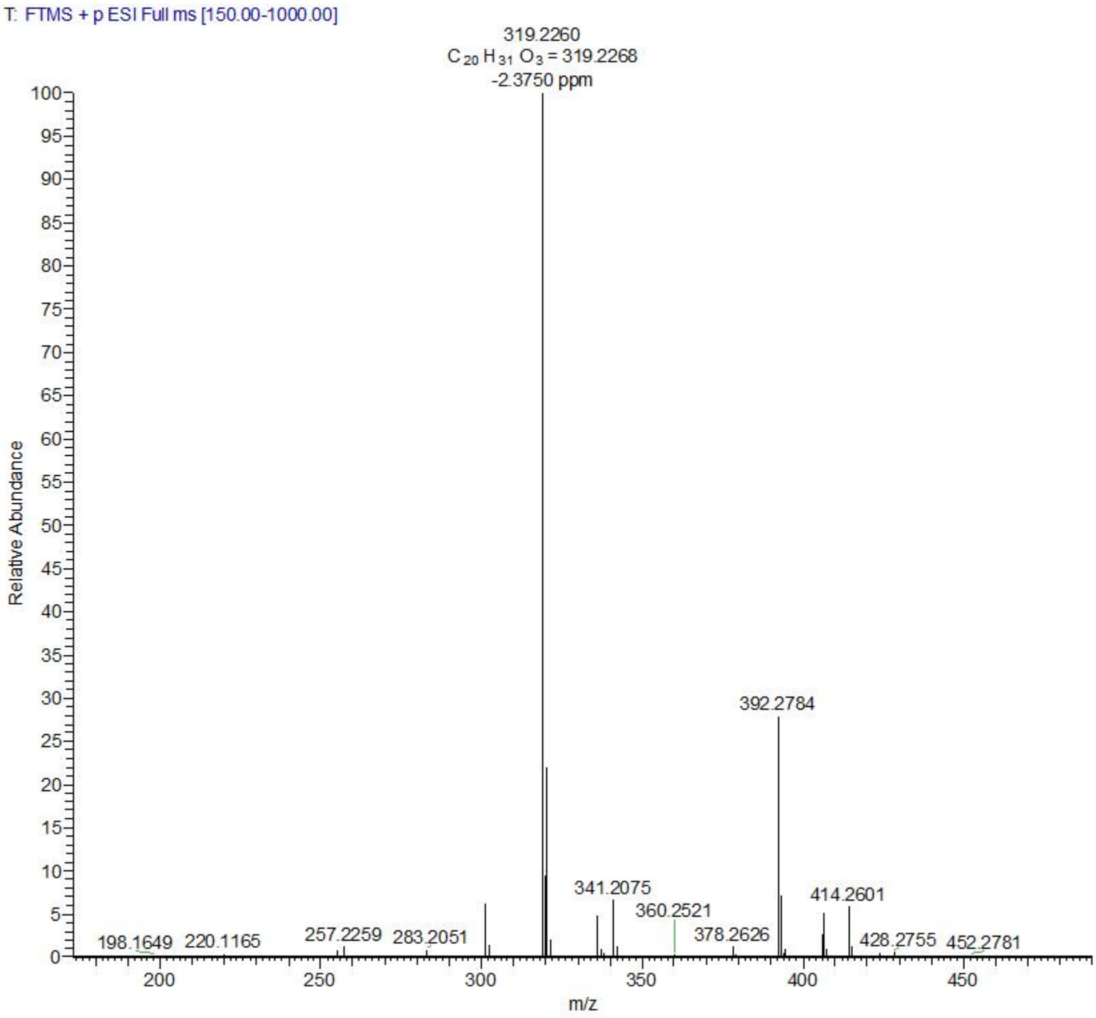

Figure S17. HRESIMS data of ghardaqenoid C (**3**).

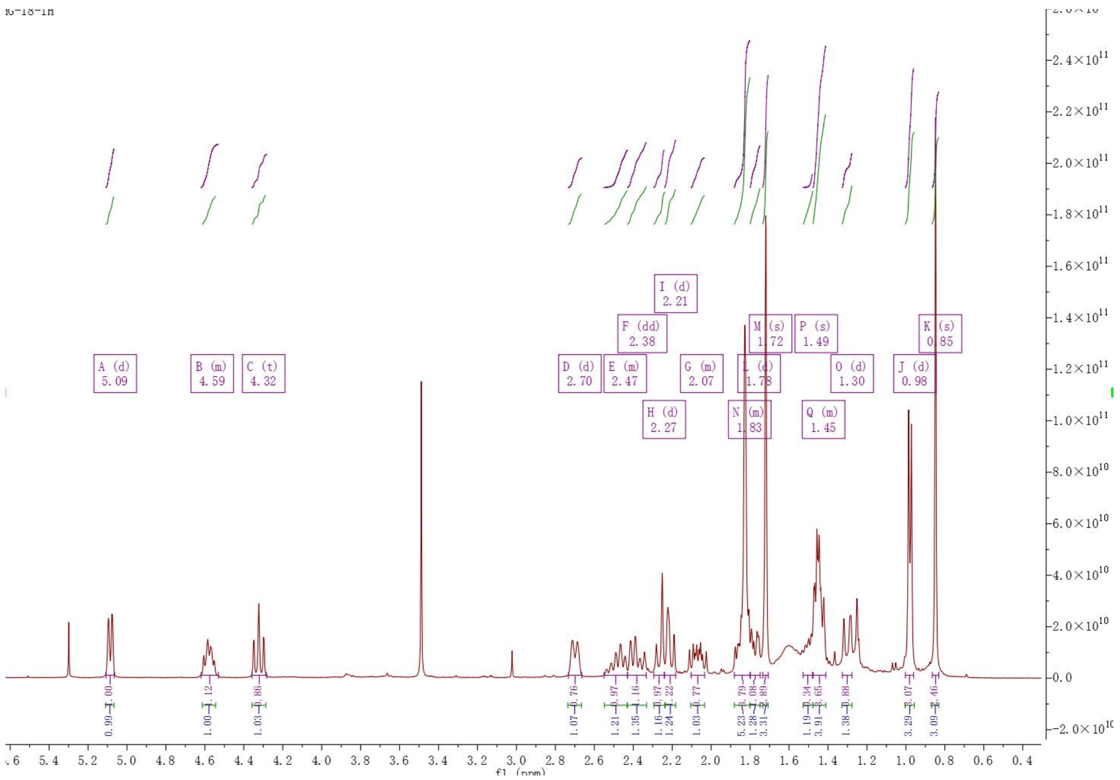

Figure S18. ^{1}H NMR spectrum of ghardaqenoid C (**3**) in $CDCl_3$ (500 MHz).

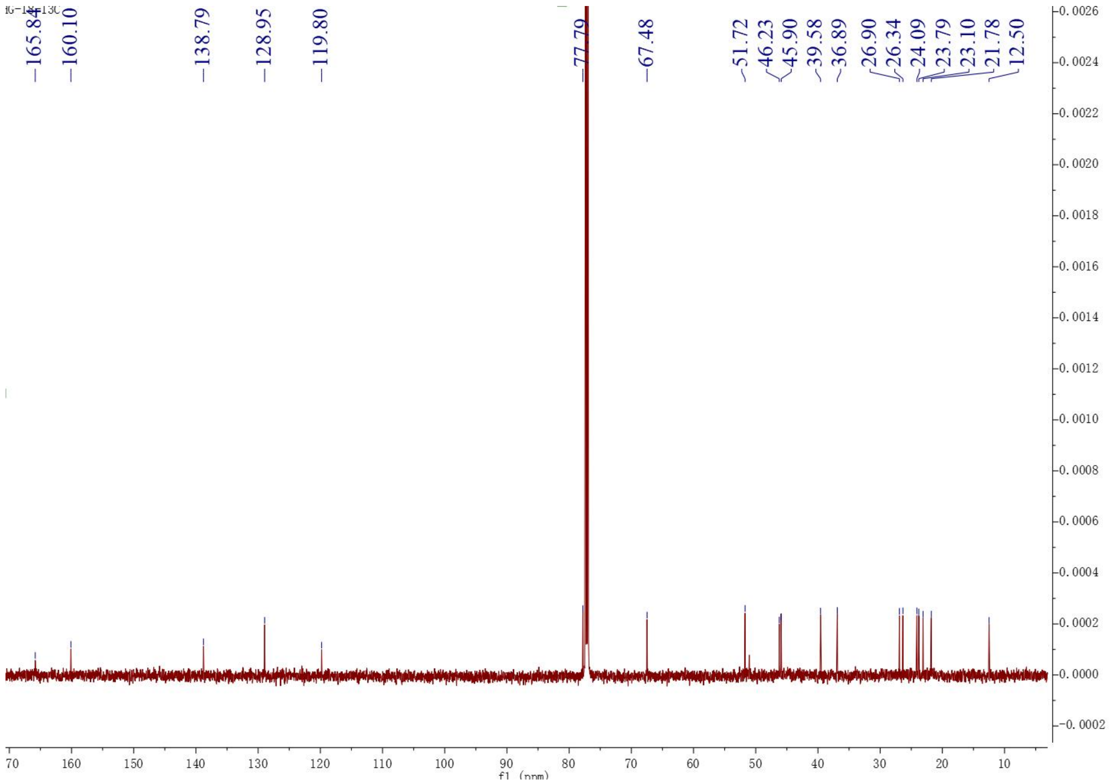

Figure S19. ^{13}C NMR spectrum of ghardaqenoid C (**3**) in $CDCl_3$ (125 MHz).

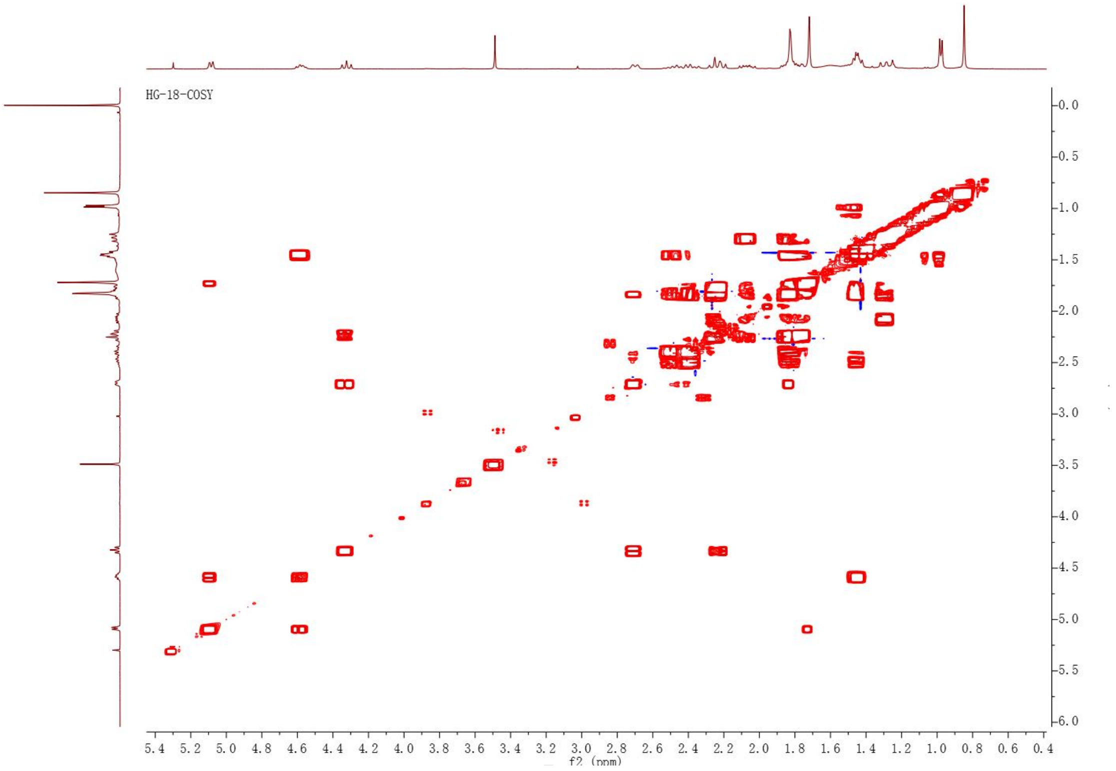

Figure S20. ^{1}H-^{1}H COSY spectrum of ghardaqenoid C (**3**) in $CDCl_3$.

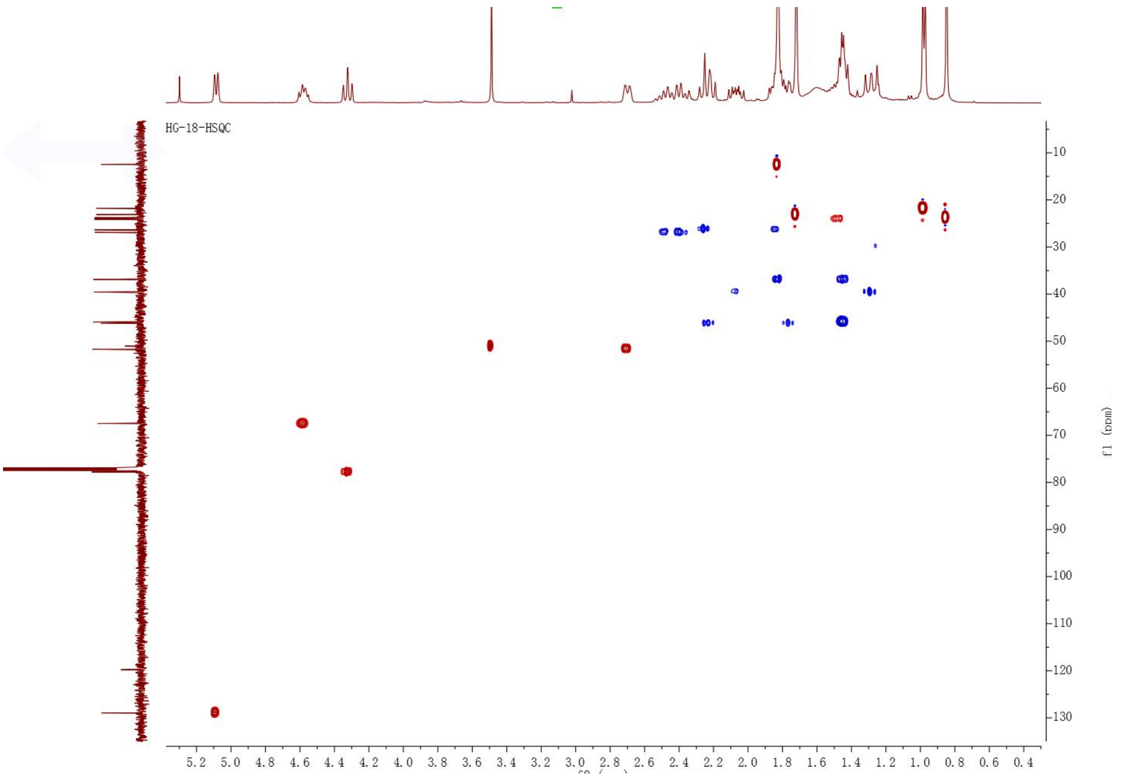

Figure S21. HSQC spectrum of ghardaqenoid C (**3**) in $CDCl_3$.

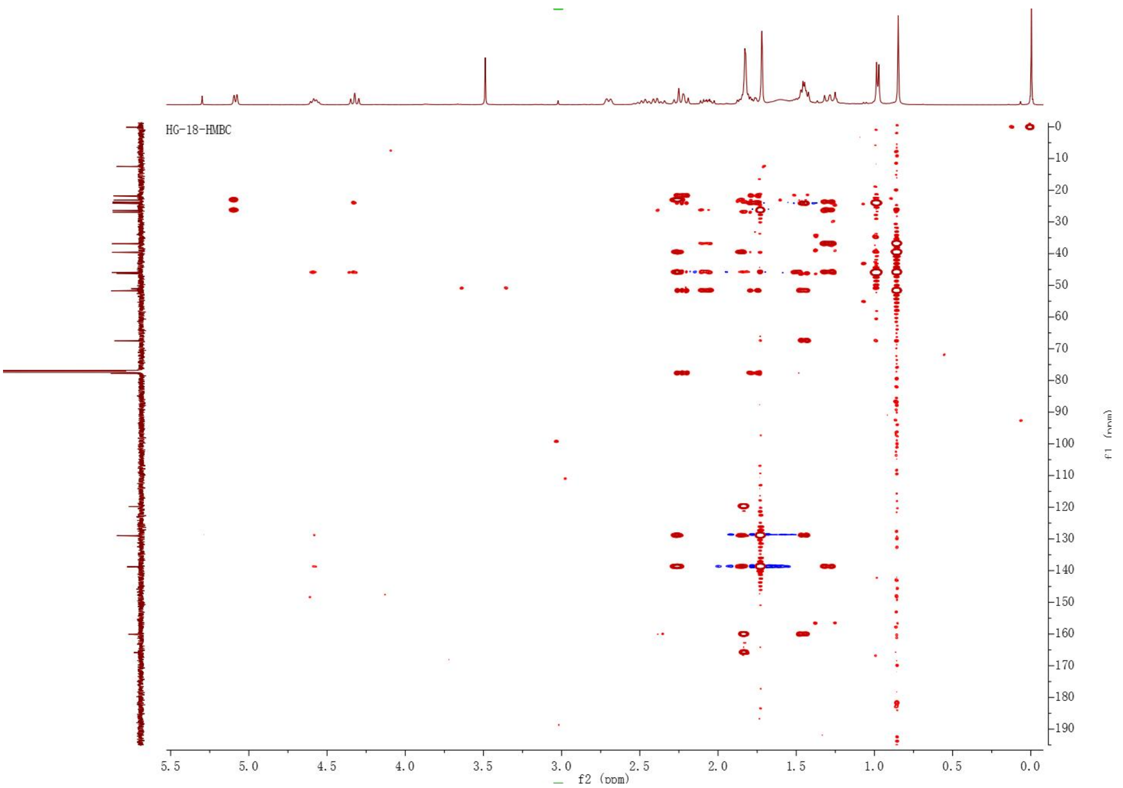

Figure S22. HMBC spectrum of ghardaqenoid C (**3**) in $CDCl_3$.

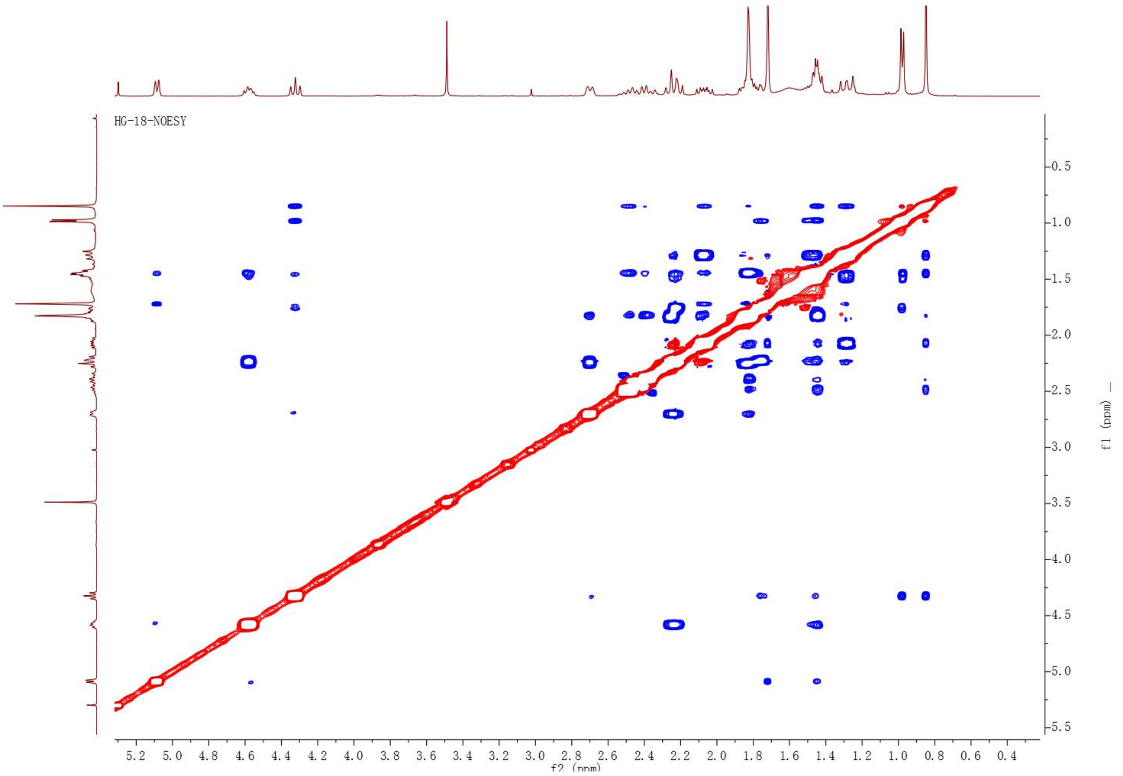

Figure S23. NOESY spectrum of ghardaqenoid C (**3**) in $CDCl_3$.

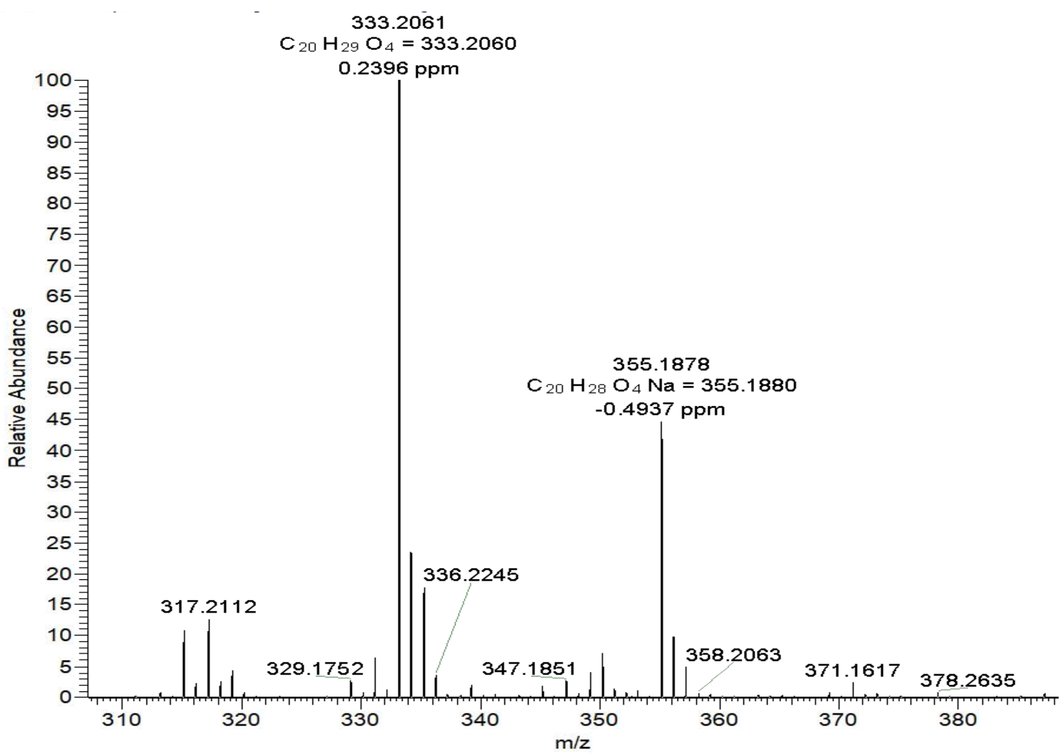

Figure S24. HRESIMS data of ghardaqenoid D (**4**).

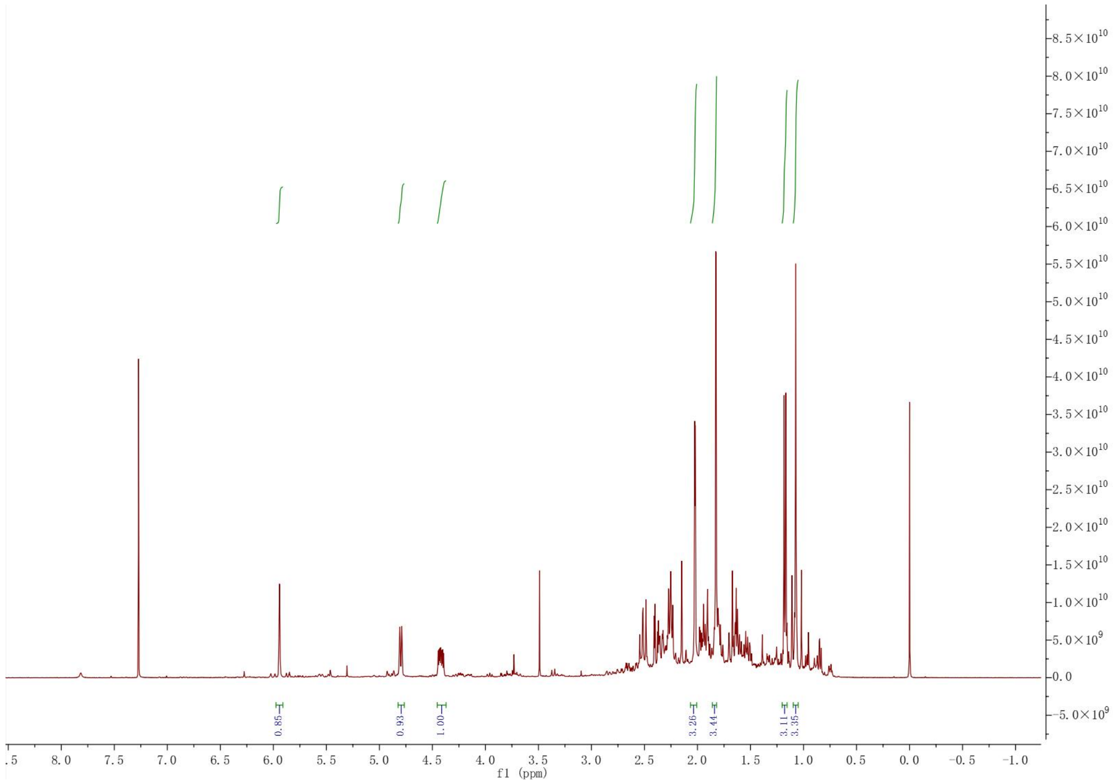

Figure S25. ^{1}H NMR spectrum of ghardaqenoid D (**4**) in $CDCl_3$ (500 MHz).

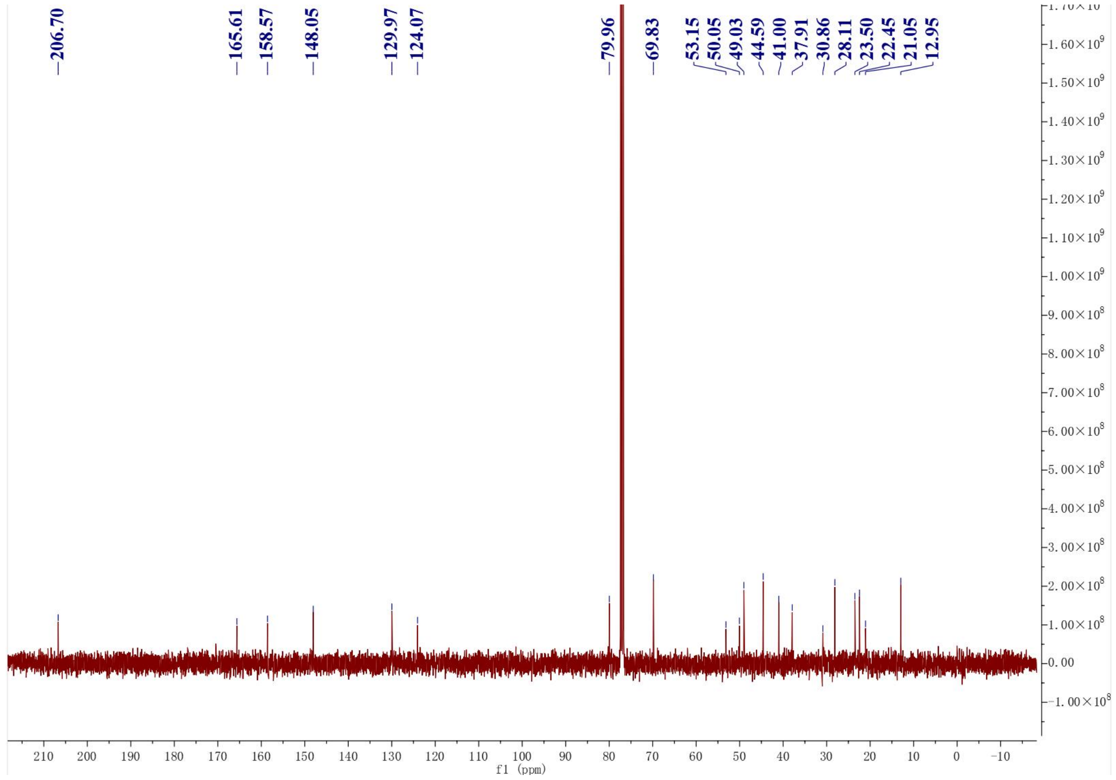

Figure S26. ^{13}C NMR spectrum of ghardaqenoid D (**4**) in $CDCl_3$ (125 MHz).

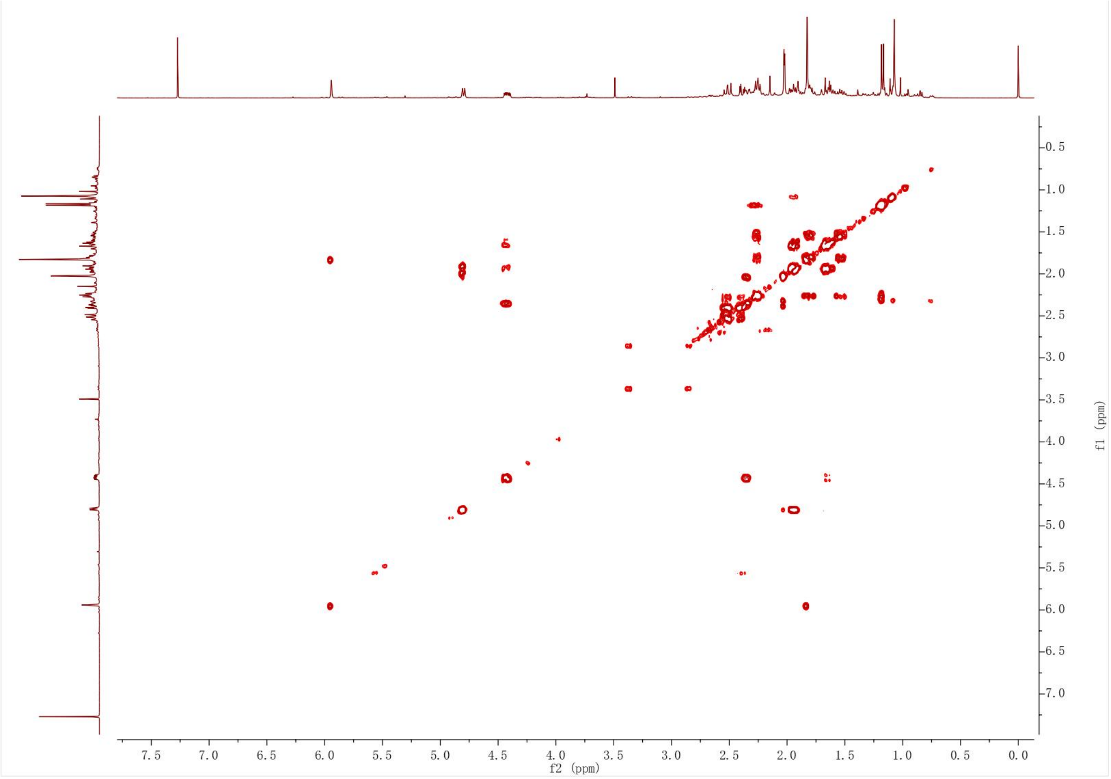

Figure S27. ^{1}H-^{1}H COSY spectrum of ghardaqenoid D (**4**) in $CDCl_3$.

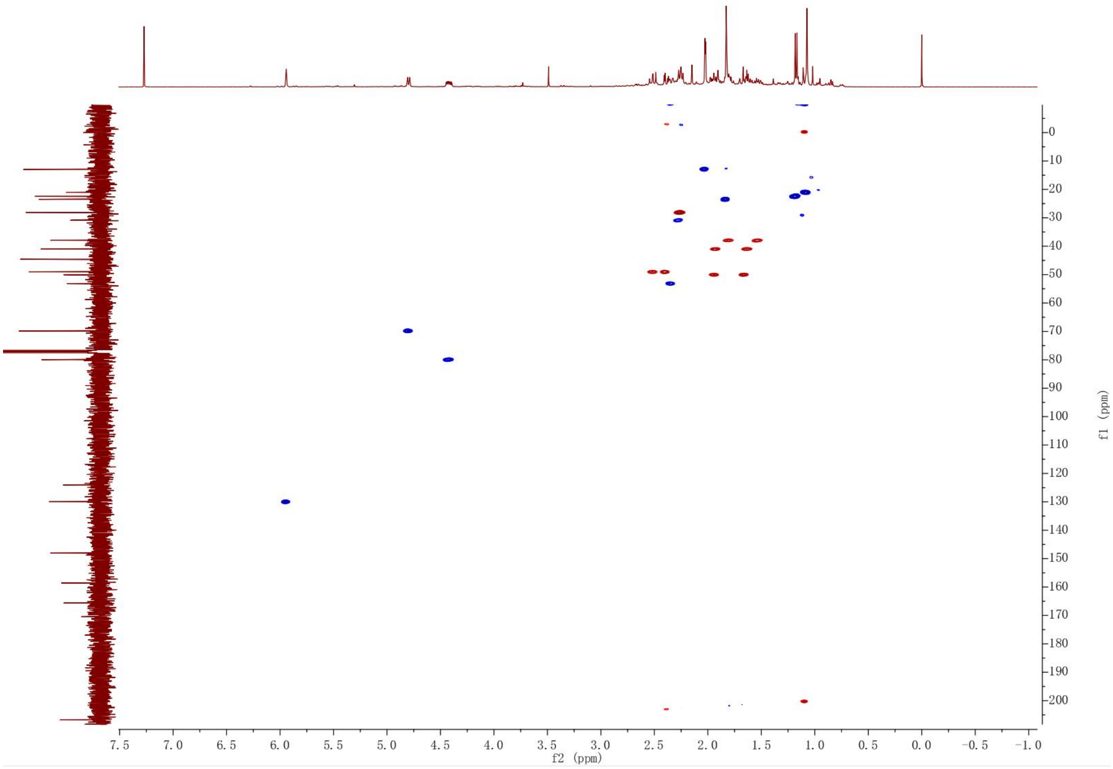

Figure S28. HSQC spectrum of ghardaqenoid D (**4**) in $CDCl_3$.

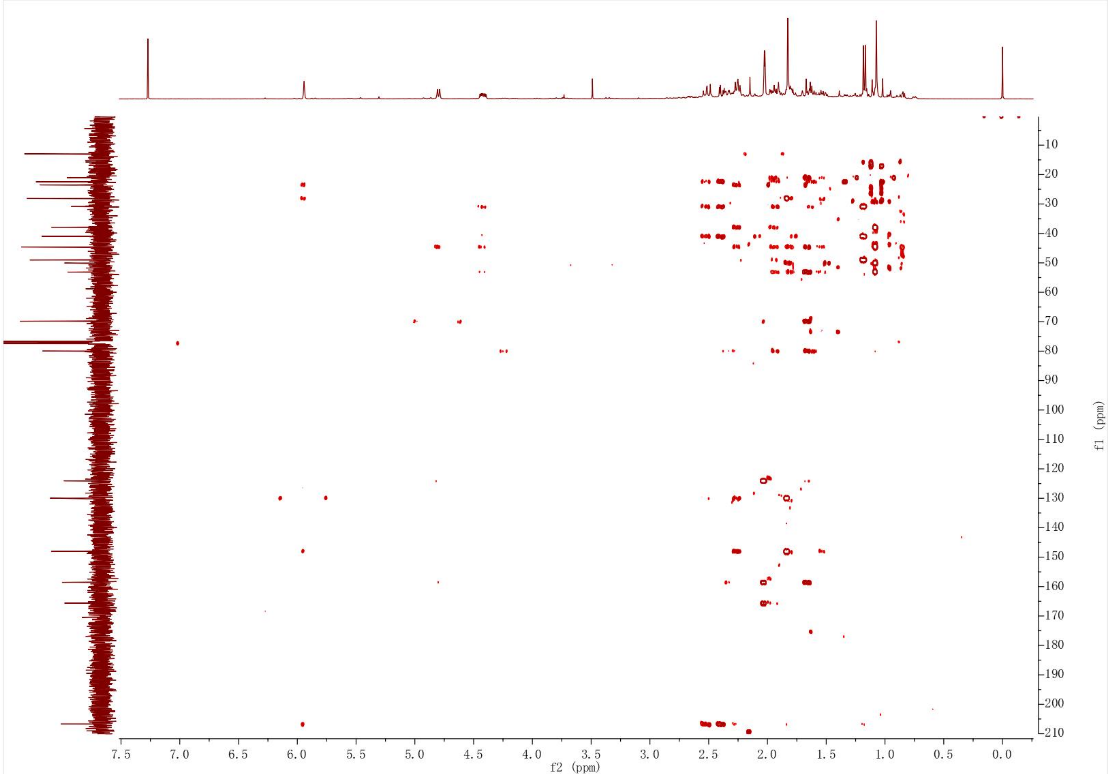

Figure S29. HMBC spectrum of ghardaqenoid D (**4**) in $CDCl_3$.

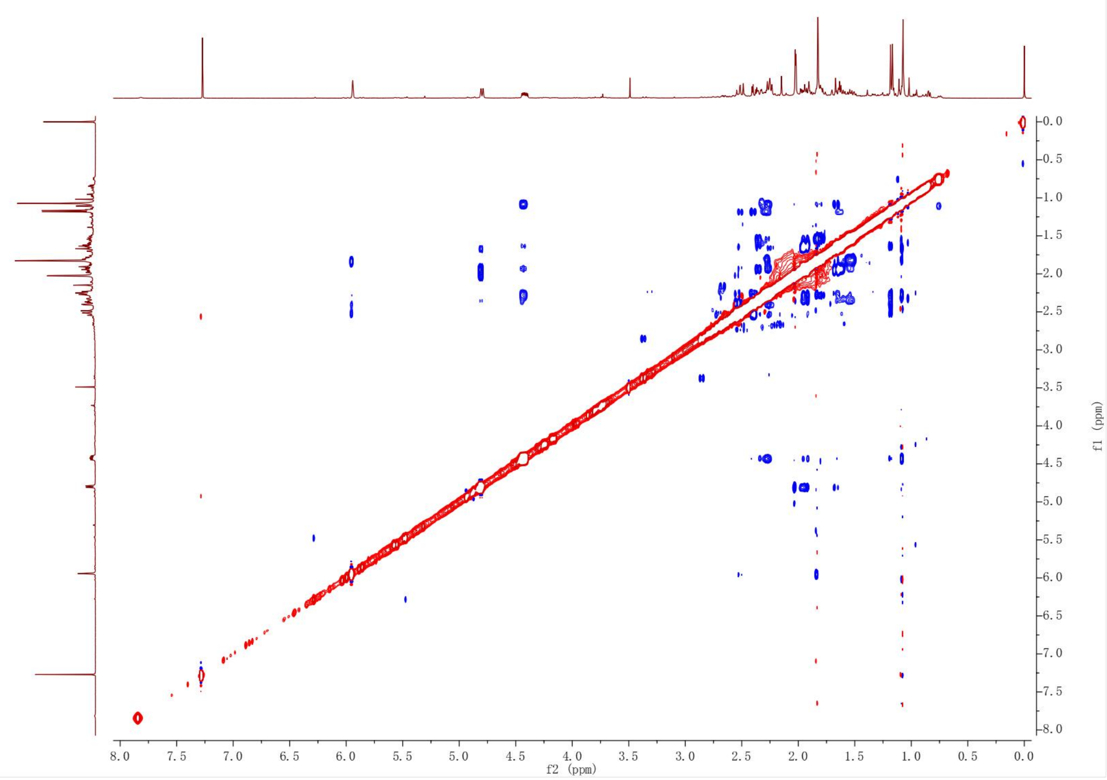

Figure S30. NOESY spectrum of ghardaqenoid D (**4**) in $CDCl_3$.

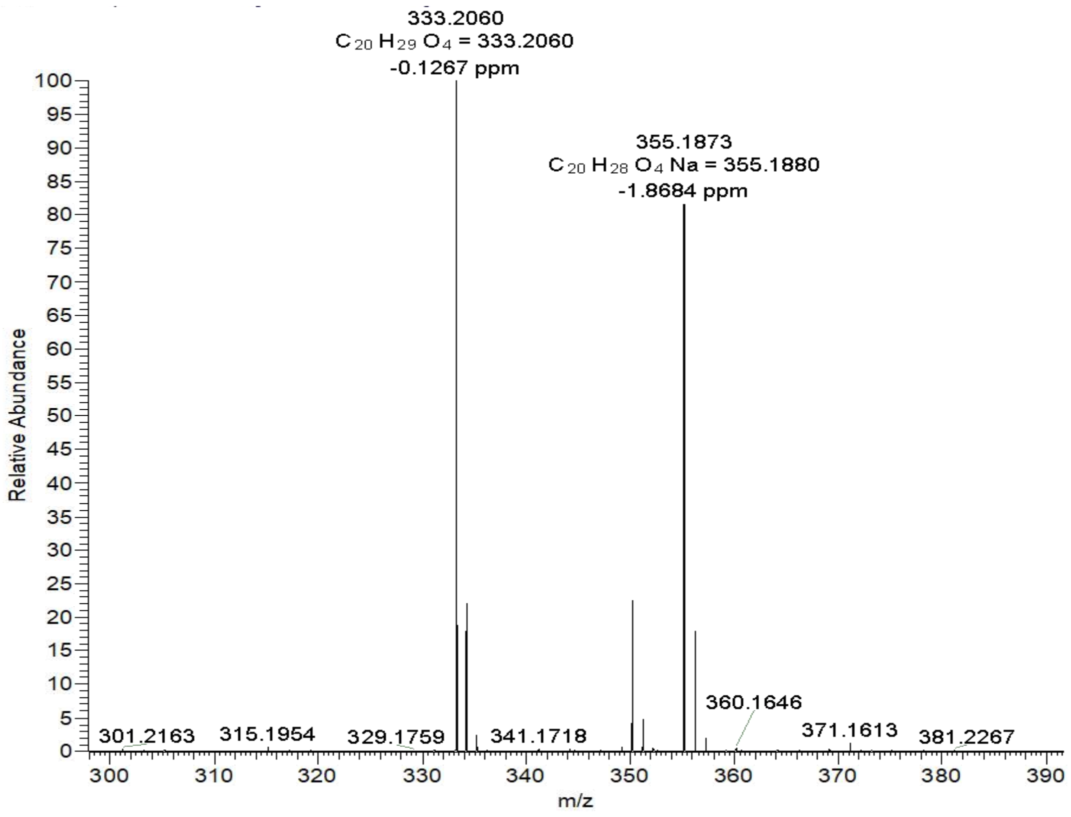

Figure S31. HRESIMS data of ghardaqenoid E (**5**).

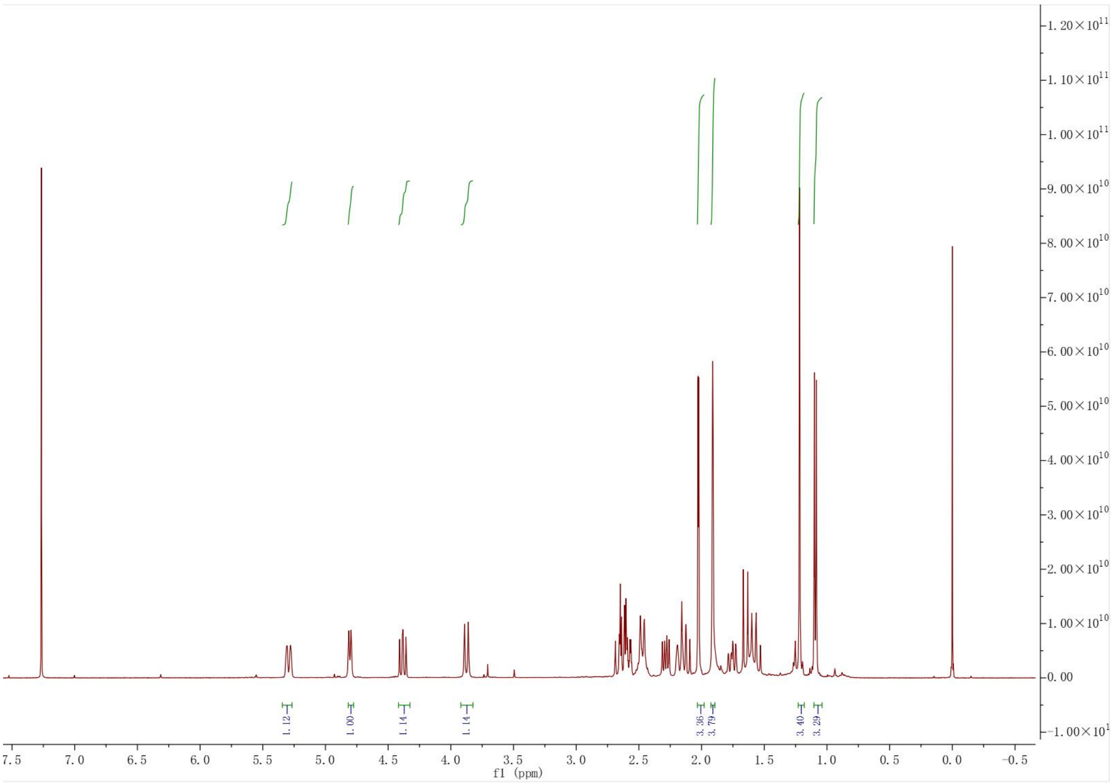

Figure S32. ^{1}H NMR spectrum of ghardaqenoid E (**5**) in $CDCl_3$ (500 MHz).

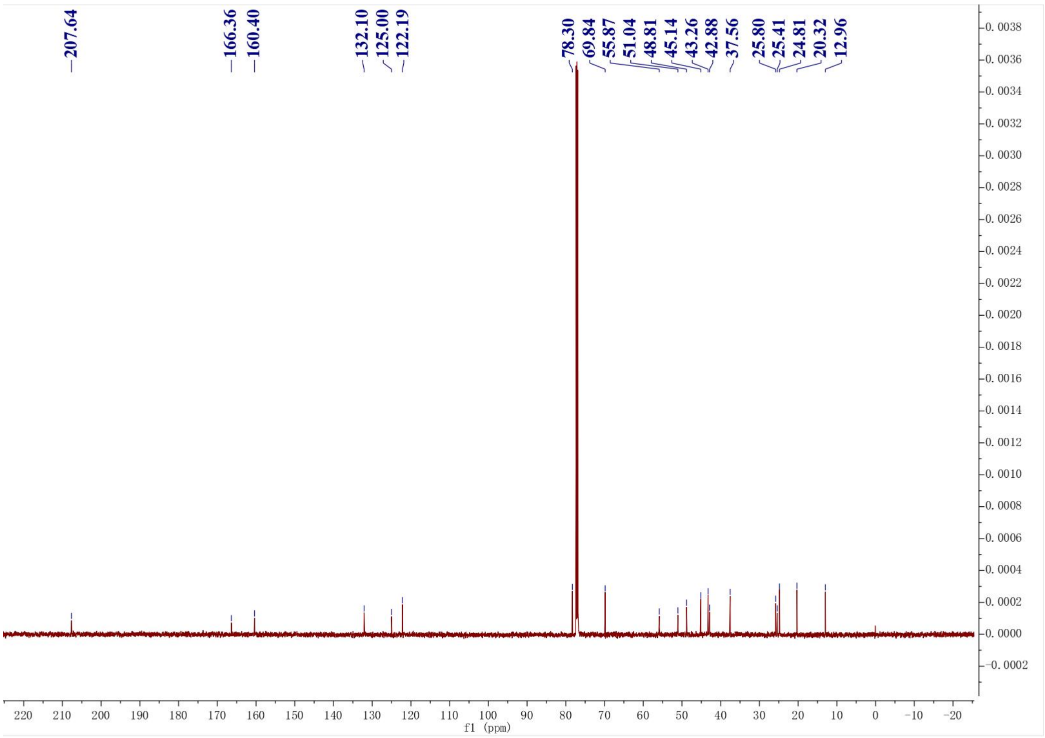

Figure S33. ^{13}C NMR spectrum of ghardaqenoid E (**5**) in $CDCl_3$ (125 MHz).

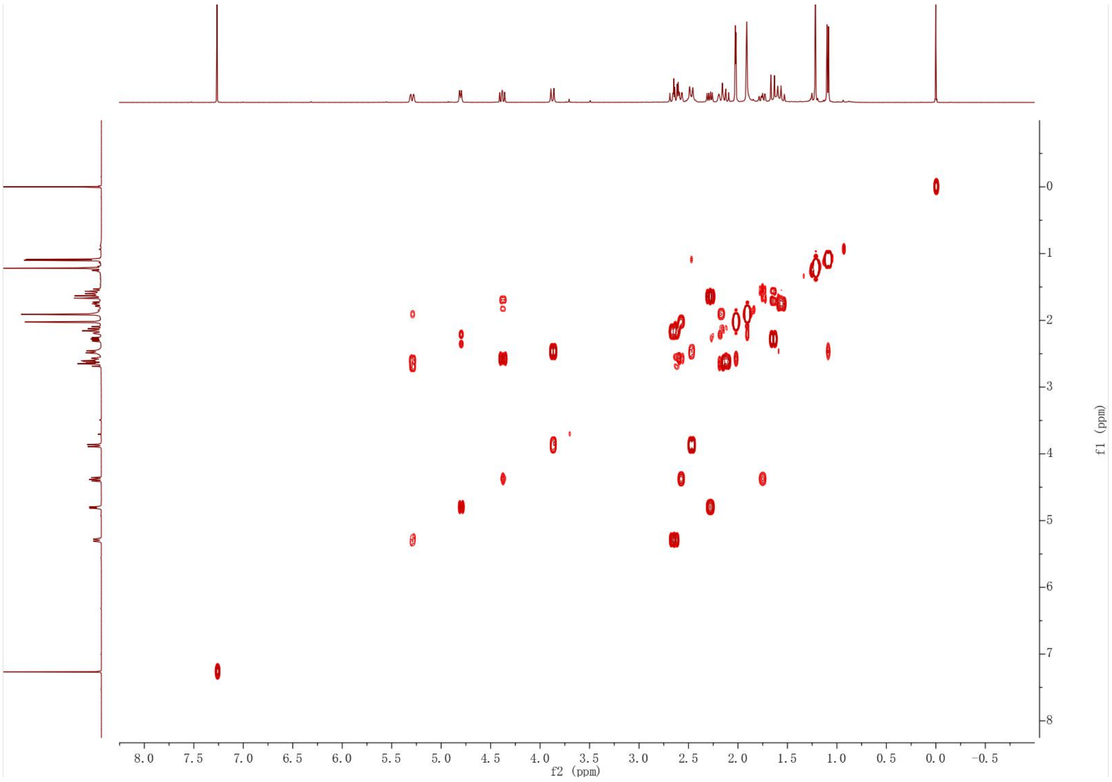

Figure S34. ^{1}H-^{1}H COSY spectrum of ghardaqenoid E (**5**) in $CDCl_3$.

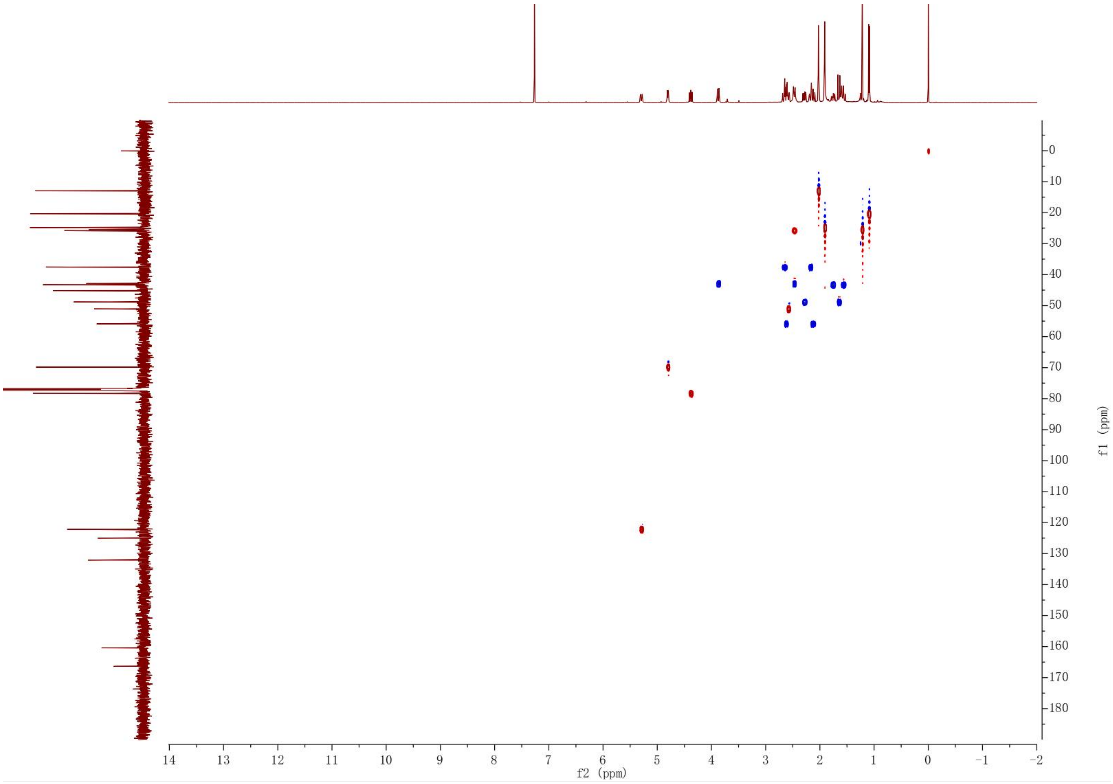

Figure S35. HSQC spectrum of ghardaqenoid E (**5**) in $CDCl_3$.

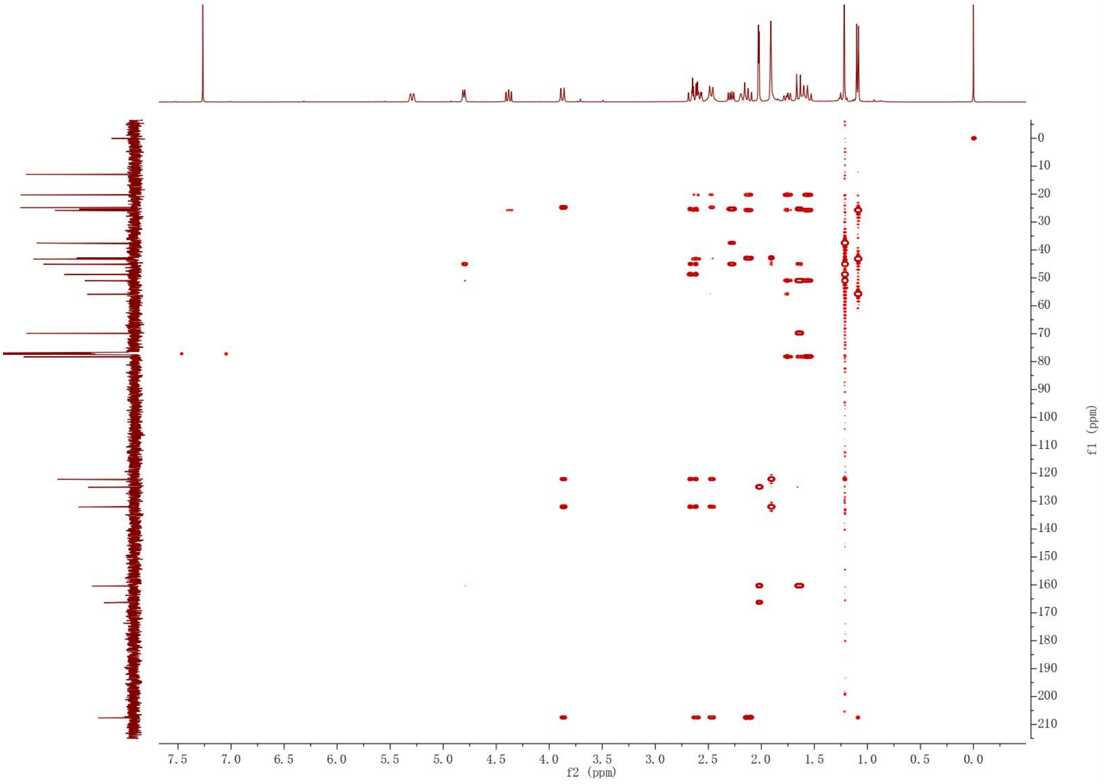

Figure S36. HMBC spectrum of ghardaqenoid E (**5**) in $CDCl_3$.

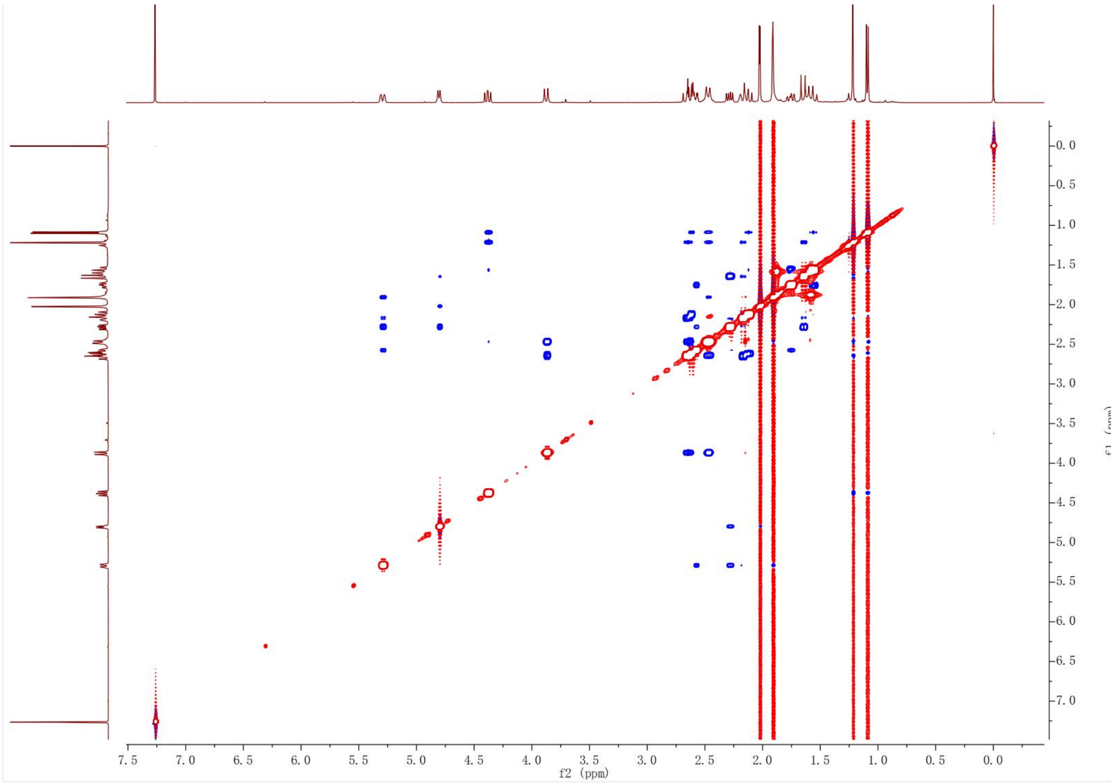

Figure S37. NOESY spectrum of ghardaqenoid E (**5**) in $CDCl_3$.

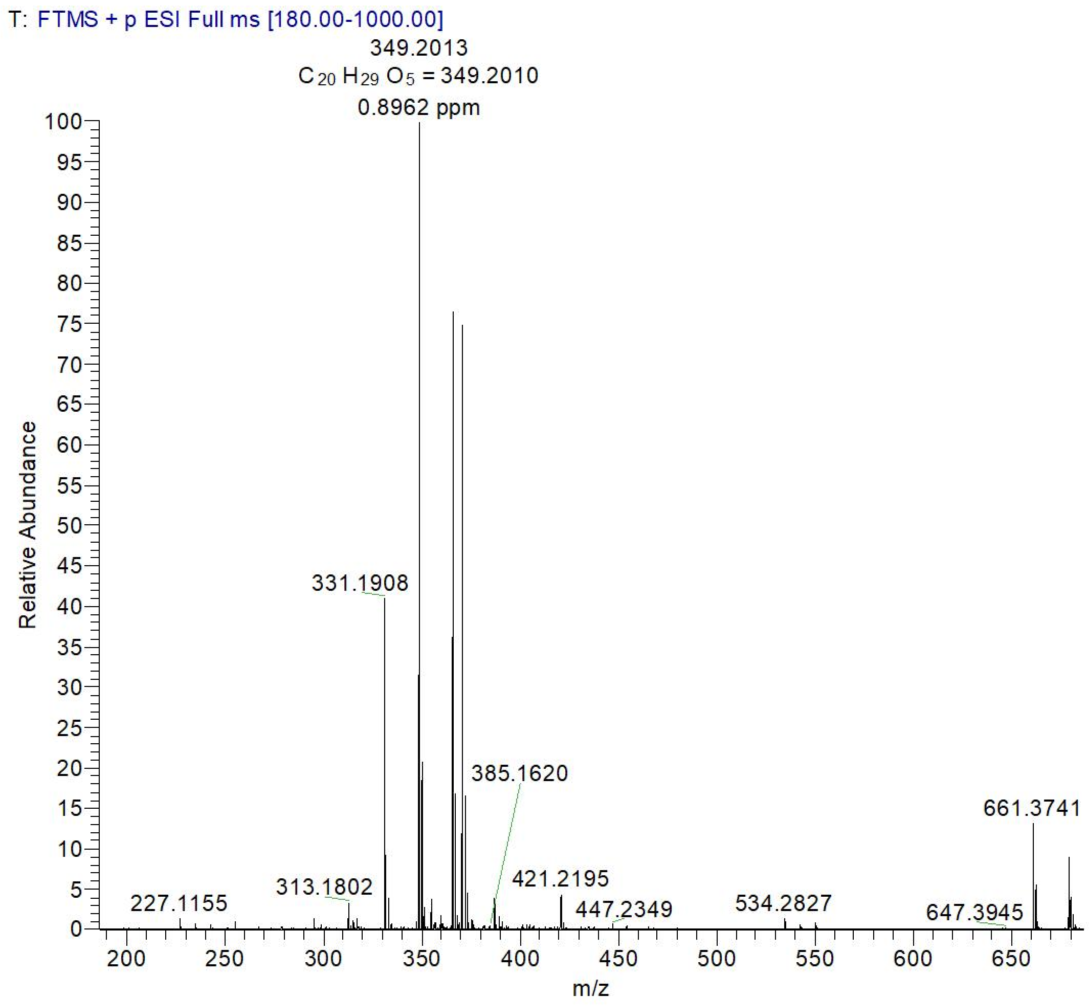

Figure S38. HRESIMS data of ghardaqenoid F (**6**).

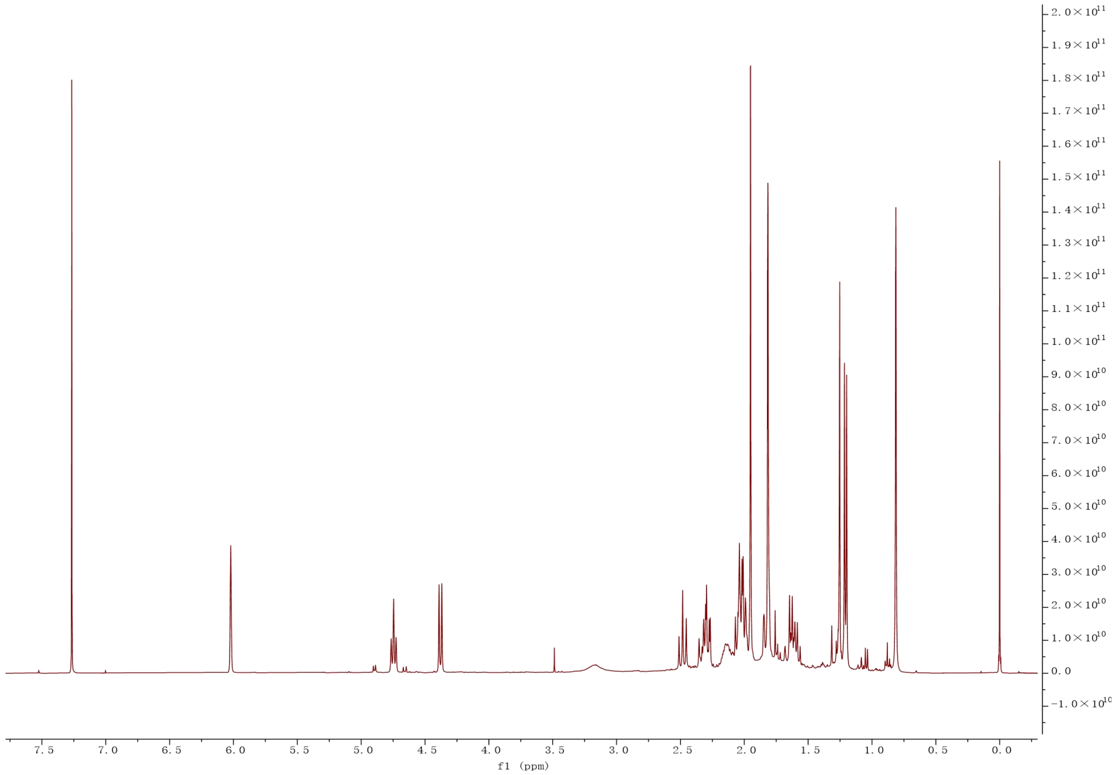

Figure S39. ^{1}H NMR spectrum of ghardaqenoid F (**6**) in $CDCl_3$ (500 MHz).

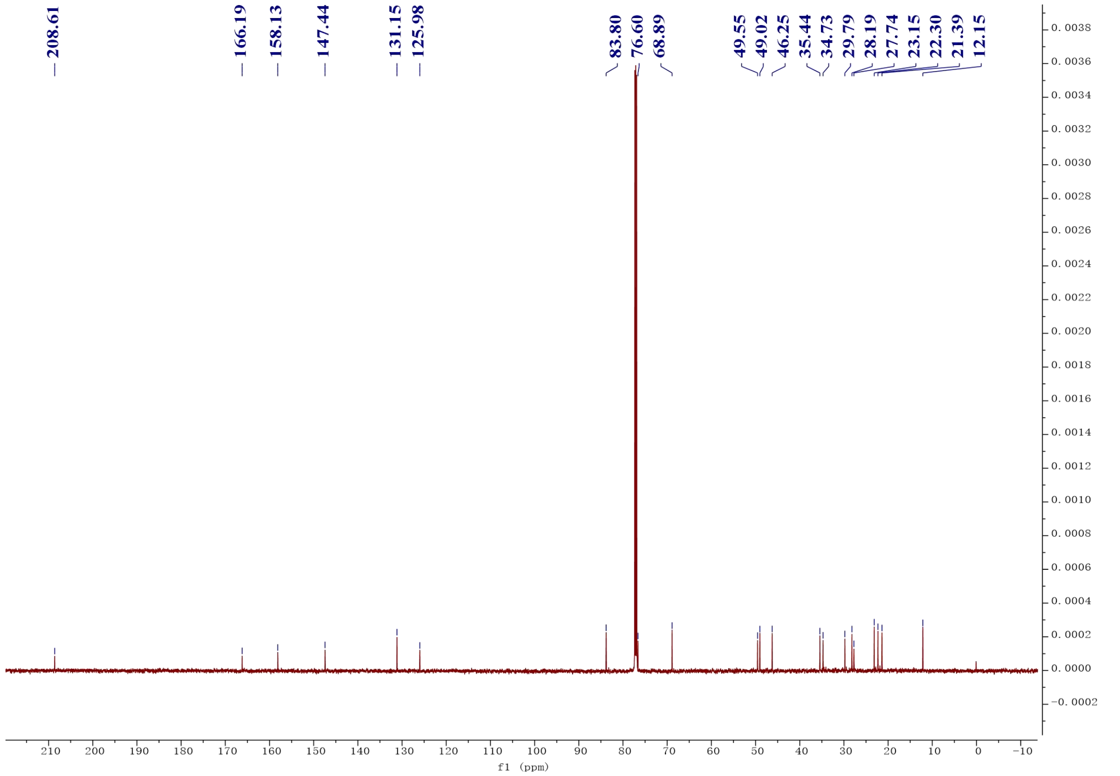

Figure S40. ^{13}C NMR spectrum of ghardaqenoid F (**6**) in $CDCl_3$ (125 MHz).

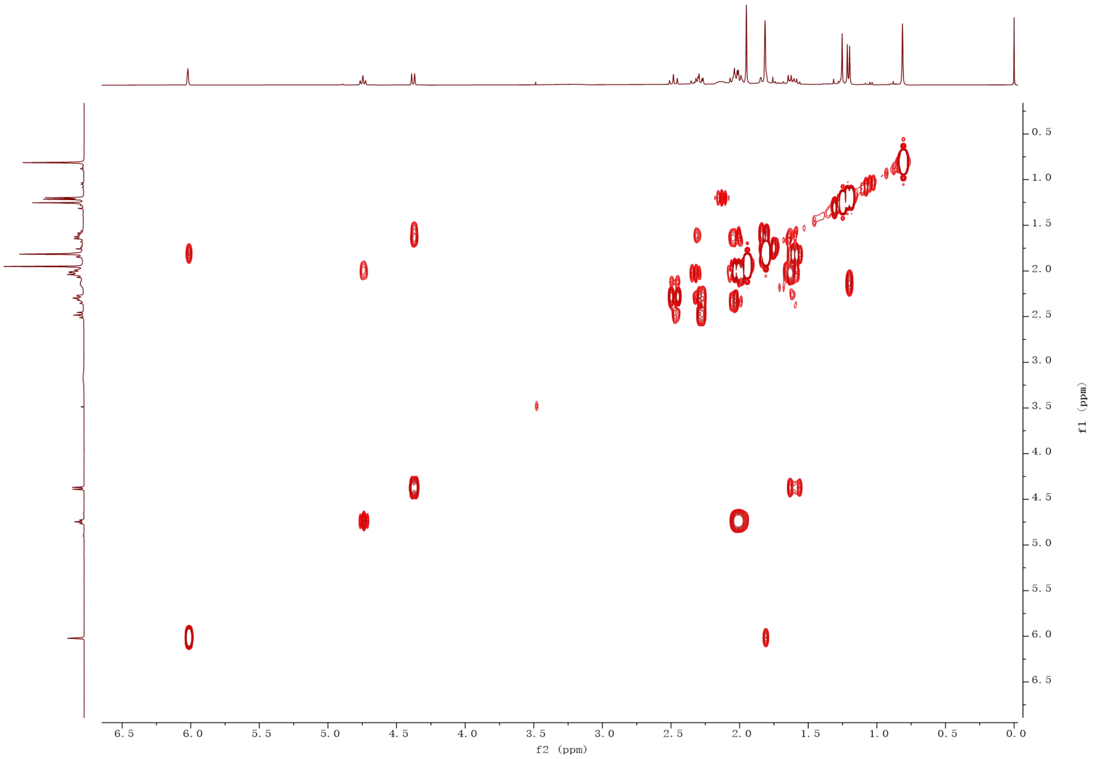

Figure S41. ^{1}H-^{1}H COSY spectrum of ghardaqenoid F (**6**) in $CDCl_3$.

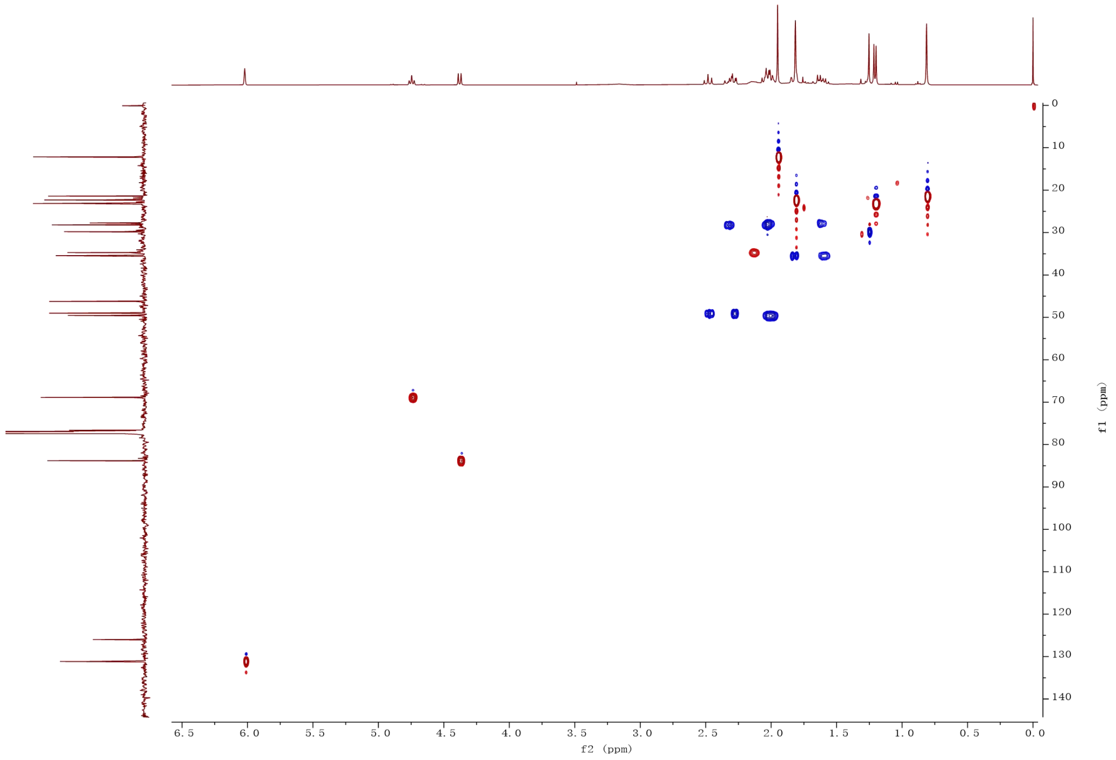

Figure S42. HSQC spectrum of ghardaqenoid F (**6**) in $CDCl_3$.

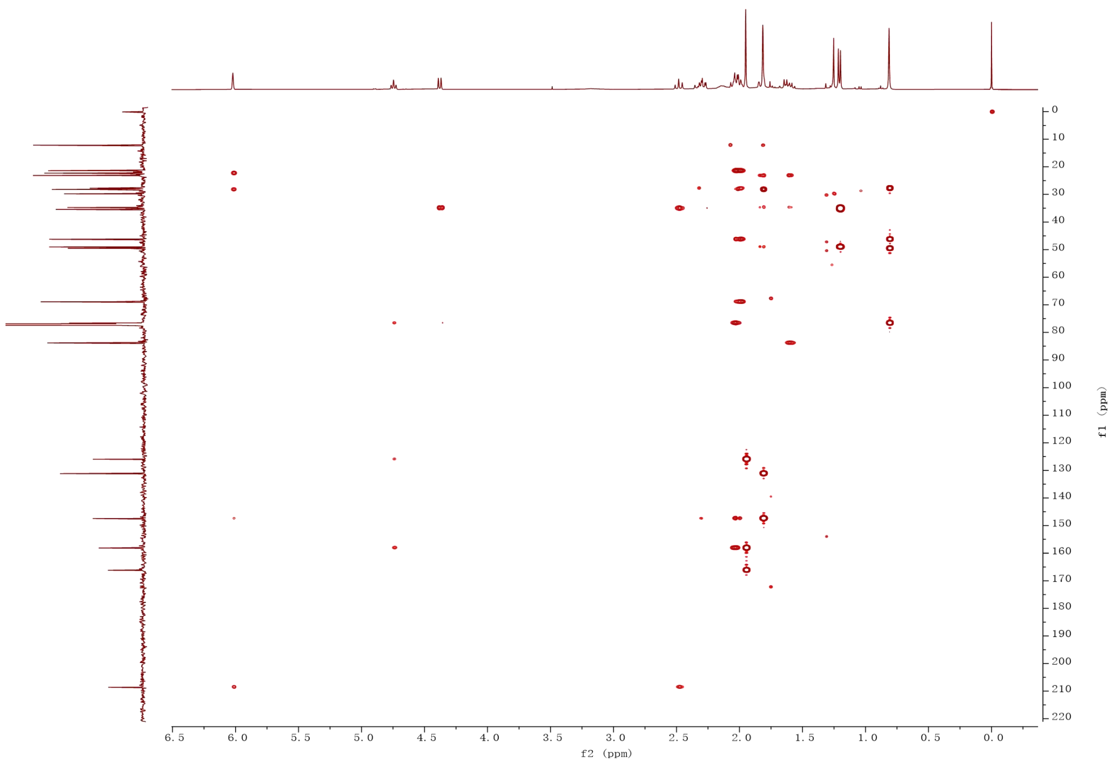

Figure S43. HMBC spectrum of ghardaqenoid F (**6**) in $CDCl_3$.

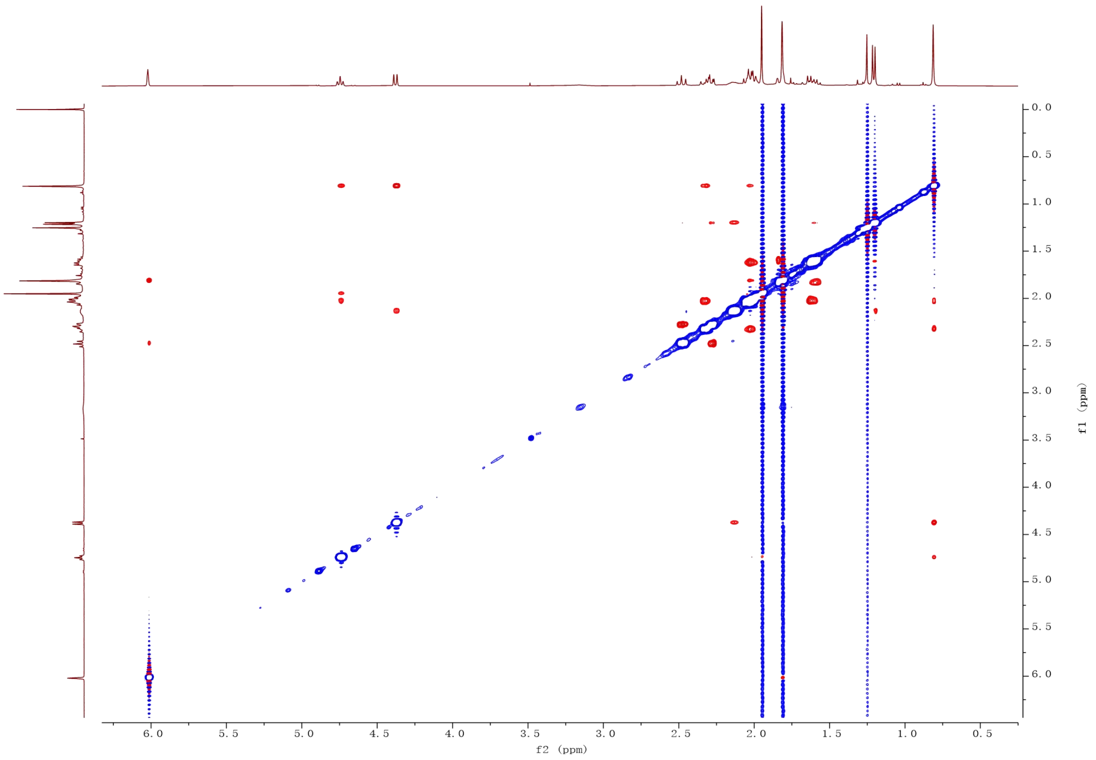

Figure S44. NOESY spectrum of ghardaqenoid F (**6**) in $CDCl_3$.

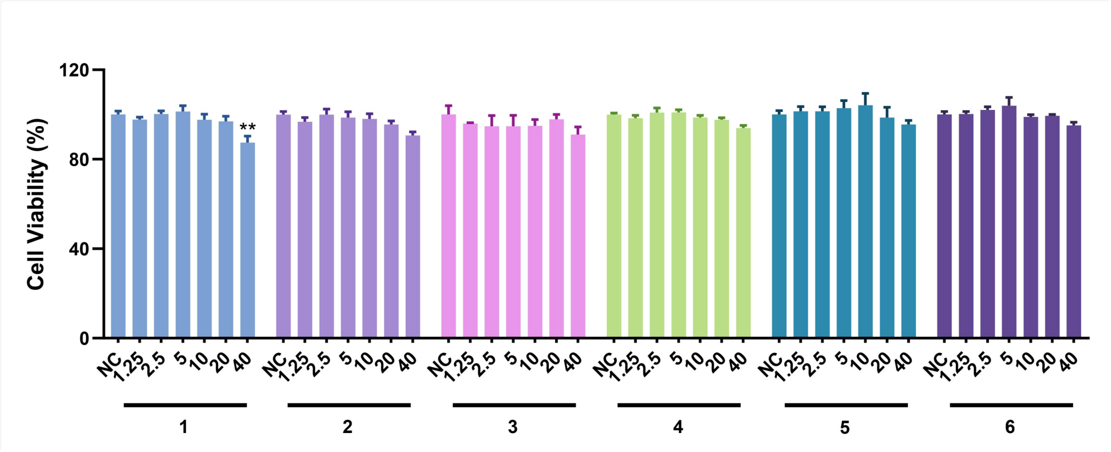

Figure S45. Cytotoxic effects of compounds on HepG2 cells.

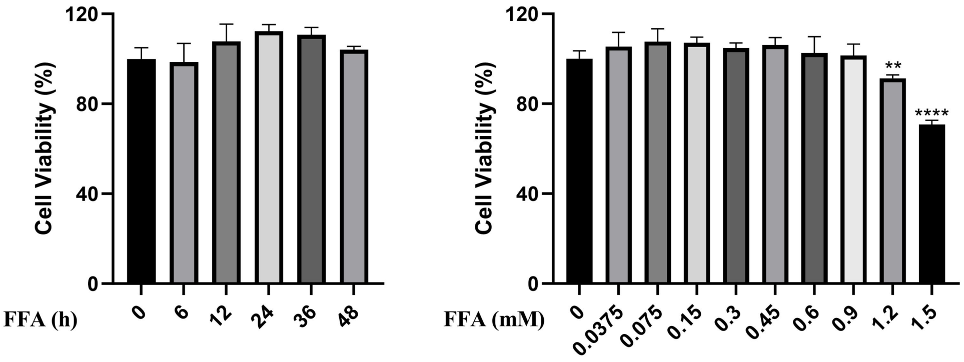

Figure S46. Effects of (Left) FFAs concentration (48 h), (Right) FFAs stimulation duration (0.45 mM FFAs; 300μM oleic acid + 150 μM palmitic acid).
