# Supplementary figures and images for: Ghardaqenoids A–F: Six New Diterpenoids from the South China Sea Soft Coral Heteroxenia ghardaqensis with Lipid-Lowering Activity via the Activation of the AMPK Signaling Pathway
Source: Mar Drugs. 2026 Jan 8;24(1):30. doi: 10.3390/md24010030 (PMC12843345; doi:10.3390/md24010030)

**Repeat1**

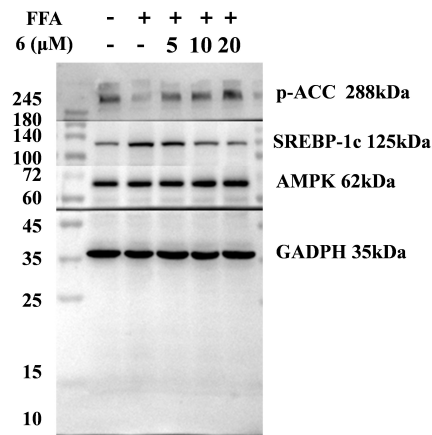

**Repeat2**

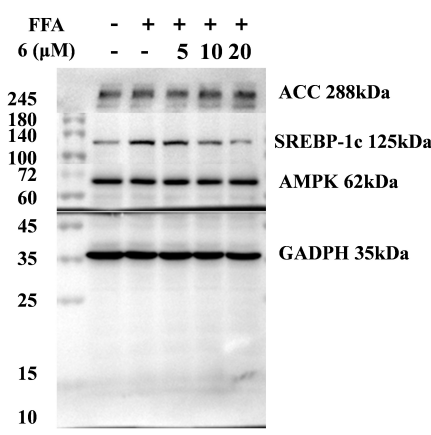

**Repeat3**

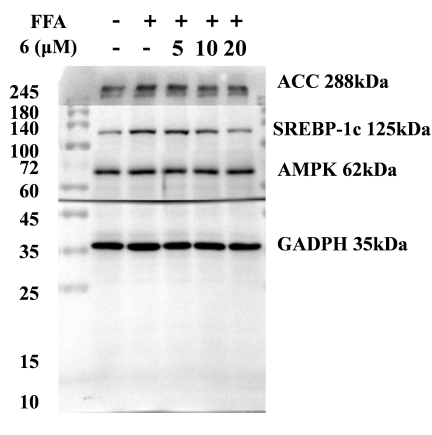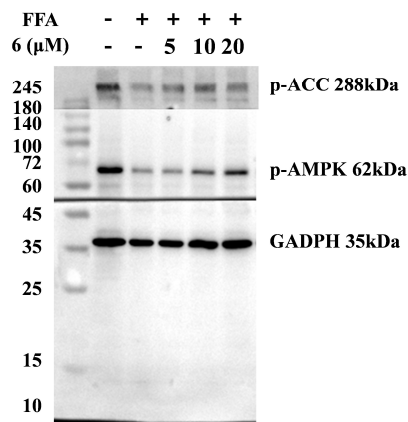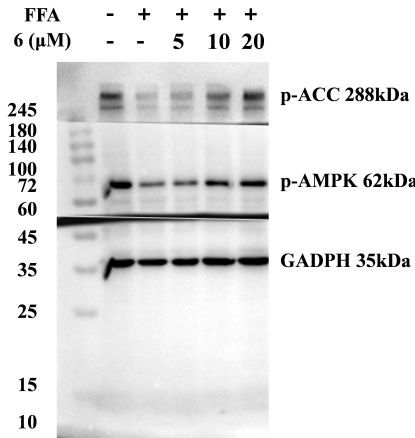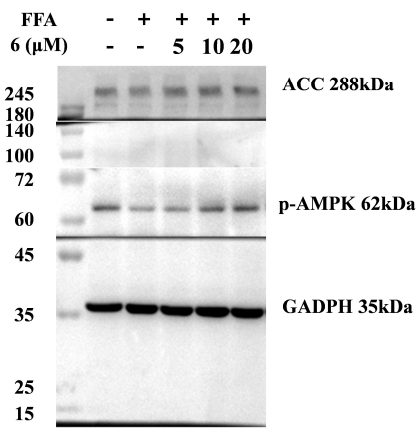

Supplement: Supplementary file 1 [file marinedrugs-24-00030-s001.zip › Original lmages for WB.pdf]
